# Cerebrovascular damage caused by the gut microbe/host co-metabolite *p*-cresol sulfate is prevented by blockade of the EGF receptor

**DOI:** 10.1080/19490976.2024.2431651

**Published:** 2024-11-24

**Authors:** Sita N. Shah, Tobias B-A. Knausenberger, Matthew G. Pontifex, Emily Connell, Gwénaëlle Le Gall, Tom A.J. Hardy, David W. Randall, Kieran McCafferty, Muhammad M. Yaqoob, Egle Solito, Michael Müller, Andrew V. Stachulski, Robert C. Glen, David Vauzour, Lesley Hoyles, Simon McArthur

**Affiliations:** a Blizard Institute, Faculty of Medicine & Dentistry, Queen Mary, University of London, London, UK; b Norwich Medical School, Biomedical Research Centre, University of East Anglia, Norwich, UK; c Department of Renal Medicine and Transplantation, Royal London Hospital, Barts Health NHS Trust, London, UK; d William Harvey Research Institute, Faculty of Medicine & Dentistry, Queen Mary, University of London, London, UK; e Dipartimento di Medicina Molecolare e Biotecnologie Mediche, Università degli Studi di Napoli Federico II, Naples, Italy; f Robert Robinson Laboratories, Department of Chemistry, University of Liverpool, Liverpool, UK; g Faculty of Medicine, Department of Metabolism, Digestion and Reproduction, Imperial College London, London, UK; h Centre for Molecular Informatics, Department of Chemistry, University of Cambridge, Cambridge, UK; i Centre for Systems Health and Integrated Metabolic Research, Department of Biosciences, School of Science and Technology, Nottingham Trent University, Clifton, Nottingham, UK; j Institute of Dentistry, Faculty of Medicine & Dentistry, Queen Mary, University of London, London, UK

**Keywords:** Blood-brain barrier, *p-cresol sulfate*, cerebrovascular disease, chronic kidney disease, gut microbiota

## Abstract

The gut microbiota-brain axis has been associated with the pathogenesis of numerous disorders, but the mechanism(s) underlying these links are generally poorly understood. Accumulating evidence indicates the involvement of gut microbe-derived metabolites. Circulating levels of the gut microbe/host co-metabolite *p*-cresol sulfate (pCS) correlate with cerebrovascular event risk in individuals with chronic kidney disease (CKD), but whether this relationship is mechanistic is unclear. We hypothesized that pCS would impair the function of the blood–brain barrier (BBB), the primary brain vasculature interface. We report that pCS exposure impairs BBB integrity in human cells *in vitro* and both acutely (≤6 hours) and chronically (28 days) in mice, enhancing tracer extravasation, disrupting barrier-regulating tight junction components and ultimately exerting a suppressive effect upon whole-brain transcriptomic activity. *In vitro* and *in vivo* mechanistic studies showed that pCS activated epidermal growth factor receptor (EGFR) signaling, sequentially activating the intracellular signaling proteins annexin A1 and STAT3 to induce mobilization of matrix metalloproteinase MMP-2/9 and disruption to the integrity of the BBB. This effect was confirmed as specific to the EGFR through the use of both pharmacological and RNA interference approaches. Confirming the translational relevance of this work, exposure of the cerebromicrovascular endothelia to serum from hemodialysis patients *in vitro* led to a significant increase in paracellular permeability, with the magnitude of permeabilization closely correlating with serum pCS, but not most other uremic toxin, content. Notably, this damaging effect of hemodialysis patient serum was prevented by pharmacological blockade of the EGFR. Our results define a pathway linking the co-metabolite pCS with BBB damage and suggest that targeting the EGFR may mitigate against cerebrovascular damage in CKD. This work further provides mechanistic evidence indicating the role of gut microbe-derived metabolites in human disease.

## Background

Chronic kidney disease (CKD) is a complex disorder affecting many aspects of physiology but is notably associated with significant neurological and cerebrovascular complications.^1^ Patients with CKD are at an increased risk of both ischemic and hemorrhagic stroke, even after correcting for coincident vascular risk factors,^2^ and tend to have worse functional outcomes post-infarct,^3^ a major unmet clinical need.^4^ Moreover, CKD is associated with both enhanced blood–brain barrier (BBB) permeability,^5^ itself a major driver of cerebrovascular disease,^6^ and the presence of microbleeds,^7^ suggesting that the cerebral vasculature is directly damaged in the condition.

A key candidate mechanism underlying this link between CKD and cerebrovascular disease is the role of the uremic toxins, particularly the conjugates of *p*-cresol. This molecule is produced by fermentation of tyrosine and phenylalanine in the colon, particularly by *Coriobacteriaceae* or *Clostridium* species.^8,9^ Luminal *p*-cresol undergoes extensive conjugation in both enterocytes^10^ and the liver,^11^ such that it is found almost exclusively as *p*-cresol sulfate (pCS, ~90% in humans) and *p*-cresol glucuronide (pCG) in the systemic circulation.^12^ Normally, these molecules are efficiently cleared by the kidneys with an effective plasma half-life of ~30 min in rodents.^13^ However, due to its high binding affinity to albumin, pCS accumulates within the plasma of individuals with compromised renal function^14^ and is poorly removed by hemodialysis,^15^ a feature of significance given that increased plasma concentrations of pCS predict mortality in individuals with CKD.^16,17^

These links between CKD and cerebrovascular disease, alongside reports from clinical studies associating plasma pCS and stroke risk^18^ and experimental work showing pCS to induce dermal microvascular leakage^19^ and aortic oxidative damage and remodeling^19,20^ led us to hypothesize that this gut microbe–host co-metabolite directly damages the cerebral vasculature, impairing the BBB function and contributing to the neurological consequences of CKD. Here, we report a clear deleterious effect of pCS upon BBB integrity, mediated through stimulation of the epidermal growth factor receptor (EGFR) and ensuing matrix metalloproteinases (MMPs) activation. Moreover, we show antagonism of the EGFR to prevent the BBB-permeabilizing actions of serum in CKD patients, offering a potential new pathway to protect against uremia-associated cerebrovascular damage linked to the actions of the gut microbiota.

## Methods

### Human serum samples

Pre-dialysis serum samples were collected from patients receiving maintenance hemodialysis at the Royal London Hospital, Whitechapel, Barts Health NHS Trust and from age-/sex-matched healthy controls undergoing evaluation for kidney donation who had previously consented for sample inclusion in the Barts Diabetic Kidney Disease Biobank, see Table 1 for summary demographic details and Supplemental Table S1 for anonymized individual details. Pre-dialysis serum samples were obtained at the end of the three-day gap between dialysis sessions, the point of maximal uremia. Estimated and measured glomerular filtration rates (GFR) are supplied for all healthy individuals.

**Table 1.** Serum donor demographics. Key demographic information for healthy and hemodialysis patient serum donor groups. Individual information is provided in Supplemental Table S1.

	Age (years)	Sex ratio	Serum urea (mg/dl)	Serum creatinine (µM)	Renal replacement therapy duration (years)
Healthy donor	49 ± 3.3	8F: 3 M	4.46 ± 0.36	82.0 ± 6.8	N/A
Hemodialysis	59 ± 6.2	4F: 6 M	19.13 ± 1.90	876.2 ± 94.7	10.6 ± 4.2

All subjects in both groups were meat-eaters who had not received systemic antibiotics for at least three months prior to inclusion in the study. Ethical approval for the inclusion in the Barts DKC Biobank was provided under REC reference no. 18/EE/0142. Approval for the current study was provided under REC reference no. 20/LO/0361. The study followed established standard operating procedures and all experiments involving human tissue conformed to the principles set out in the World Medical Association Declaration of Helsinki. All serum samples were de-complemented by incubation at 56°C for 20 min prior to use.

### Animals

Wild-type, male, specific pathogen-free, C57Bl/6J mice (Charles River UK Ltd., Margate, UK) were used for all experiments, and were aged between 7 and 8 weeks at the start of experiments. All experiments were approved by the QMUL Animal Welfare and Ethical Review Board (acute studies) or the University of East Anglia Animal Welfare and Ethical Review Body (long-term studies) and were performed in accordance with the ARRIVE guidelines and the UK Animals (Scientific Procedures) Act, 1986, under UK Home Office Project Licenses PFA5C4F4F and PP9417531.

### Animals: acute studies

Mice were housed in groups of five per cage in a room with controlled lighting (lights on 08.00–20.00 h) and temperature (21–23 °C) and with standard laboratory chow (5058, LabDiet, St. Louis, USA) and water available *ad libitum*. Mice were acclimatized to the animal facility environment for one week prior to experimentation. Three experiments were performed. 1) Mice (*n* = 5-6) were injected i.p. with 10 mg/kg pCS (CAS 91,978-69-7) in 100 µl 0.9% saline vehicle (a dose chosen to approximately double circulating free pCS concentrations,^21^ which was allowed to circulate for 2 h or 6 h before animals were killed by transcardial perfusion with ice-cold 0.1 M phosphate-buffered saline (PBS) under deep sodium pentobarbital anesthesia (50 mg/kg, i.p.). 2) Mice (*n* = 5–6) were injected with erlotinib (50 mg/kg, i.p., CAS 183,321-74-6) 1 h prior to pCS (10 mg/kg, i.p.), which was allowed to circulate for 2 h prior to killing by transcardial perfusion as above. 3) Mice (*n* = 5-6) were injected with SB-3CT (10 mg/kg i.p.; CAS 292,605-14-2) 1 h prior to pCS (10 mg/kg, i.p.), which was allowed to circulate for 2 h prior to killing by transcardial perfusion as above. In all cases, whole blood was collected and permitted to coagulate for 30 min at room temperature, prior to centrifugation at 800 *g* for separation of serum, and brains were rapidly removed and bisected. For studies of tracer extravasation (experiments 1, 2 and 3), half-brains were collected into 0.1 M PBS at 4°C for later analysis as described below. For RNAseq studies (experiment 1), half-brains were stored in RNAlater (ThermoFisher Scientific Ltd., UK) at 4°C overnight, and then frozen at −80°C for later RNA extraction as described below. For immunohistochemical analysis (experiment 2), half-brains were fixed by immersion in 4% formaldehyde in 0.1 M PBS at 4°C for 24 h, after which they were embedded in paraffin wax. In all cases, analysis was performed blinded to original groupings.

### Animals: long-term studies

Mice were housed in groups of four per cage in a room with controlled lighting (lights on 08.00–20.00 h) and temperature (21–23 °C) and with standard lab chow (5058, LabDiet, St. Louis, USA) and water available *ad libitum*. Mice were acclimatized to the animal facility environment for two weeks prior to experimentation. Animals were anesthetized by isoflurane inhalation and subcutaneously implanted with osmotic minipumps (model 2004, Alzet Cupertino, CA, USA) previously loaded with either 30 mg/ml pCS (*n* = 7; Tokyo Chemical Industry UK Ltd., Oxford, UK) or saline vehicle (*n* = 10), releasing at 0.25 µl/h for 4 weeks. One week following minipump implantation, mice underwent behavioral assessments for, sequentially, open field behavior, Y-maze performance, and novel object recognition test as described below. BBB permeability was then assessed using sodium fluorescein, and mice were killed by transcardial perfusion with ice-cold 0.1 M PBS under deep sodium pentobarbital anesthesia (50 mg/kg, i.p.). Serum and brains were collected as described above, with left hemispheres prepared for analysis of sodium fluorescein extravasation and right hemispheres prepared for immunohistochemical analysis. In all cases, analysis was performed blinded to original groupings.

### In vivo BBB permeability analysis

Mice were injected i.p. with 100 µl of a 2% w/v solution of Evans blue tracer in 0.9% saline (Merck Ltd, Poole, UK) or i.v. with 200 µl of a 2% w/v solution of sodium fluorescein in 0.9% saline (Merck Ltd., UK). The tracer was permitted to circulate for 1 h before animals were killed by transcardial perfusion with 0.1 M PBS under deep sodium pentobarbital anesthesia as described above. Left hemispheres were collected and homogenized by maceration in 0.1 M PBS, and suspended macromolecules were precipitated by incubation with 60% trichloroacetic acid. The tracer content of the resulting supernatants was assessed using a CLARIOstar spectrophotometer (BMG Labtech GmbH, Germany), set to read absorbance at 620 nm for Evans blue or fluorescence at 516 nm for sodium fluorescein alongside a standard curve of defined concentrations of the tracer in the same buffer. Brain tracer content was expressed as µg of dye/mg of brain tissue, normalized to circulating serum concentrations.

### RNAseq analysis

Whole-brain total RNA was extracted and samples (*n* = 5 pCS, *n* = 5 saline control) were sent to Macrogen Inc. (Republic of Korea) for RNAseq analysis, followed by in-house processing of raw RNAseq sequence data as described previously.^22^ DESeq2 v1.34.0^23^ was used to identify significantly (*p* < 0.05, Benjamini-Hochberg) differentially expressed genes in the dataset. Entrez gene identifiers were converted to gene symbols using *Mus musculus* annotations downloaded from NCBI on 12 October 2022. Enrichr^24^ was used to perform Gene Ontology analysis. SPIA v2.48.0^25^ was used to identify KEGG pathways inhibited or activated in mouse brain samples exposed to pCS. KEGGgraph v1.56.0^26^ was used to generate the topological network from the log_2_ fold change values for the significantly differentially expressed genes and KEGG xml files for the *Mus musculus* pathways of interest. igraph v1.3.5^27^ was used to generate summary network statistics. Raw RNAseq data have been deposited with ArrayExpress under accession number E-MTAB-12326.

### Immunohistochemistry

Paraffin-embedded brains were sectioned (5 µm) using a rotary microtome and collected onto glass microscope slides. Following deparaffinisation, sections were immunostained essentially as described previously.^22^ Primary antibodies used were MMP2: rabbit anti-human, 1:1000 Insight Biotechnology Ltd., UK, MMP9: mouse anti-human, 1:50, Santa Cruz Biotechnology Inc, USA, phosphotyrosine-21 ANXA1: rabbit anti-human, 1:1000,^28^ ZO-1: rabbit anti-human 1:100, Thermofisher Scientific, UK. Secondary antibodies used were horseradish peroxidase-conjugated goat anti-rabbit or anti-mouse 1:500, both Stratech Scientific, UK, AF488-conjugated goat anti-rabbit or anti-mouse 1:500, both Thermofisher Scientific, UK. Sections were counterstained with hematoxylin or 50 ng/ml 4′,6-diamidino-2-phenylindole (DAPI) as appropriate and mounted for microscopic examination. Brightfield images were captured using a using a Nikon Eclipse 80i Stereology Microscope fitted with an Optronics Camera, using a 40× objective (NA: 0.75 mm, working distance: 0.66 mm). Fluorescence images were captured with an LSM880 confocal laser scanning microscope (Carl Zeiss Ltd., Cambridge, UK) fitted with 405, 488 and 561 nm lasers and an ×63 oil immersion objective lens (NA: 1.4 mm, working distance: 0.17 mm), using ZEN imaging software (Carl Zeiss Ltd., UK). All images were analyzed with ImageJ 1.53 k software (National Institutes of Health, USA).

### Behavioural analyses

Behavioural tests were performed in the order they are introduced below. Apparatus was cleaned using 70% ethanol upon completion of each trial, eliminating any residual odor.

An open field test (OFT) was conducted as previously described.^22^ Briefly, mice were placed in the center of the OFT, a gray 50 × 50 × 50 cm apparatus illuminated with low lux (100 lux) lighting. Total travel distance and time spent in the center of the field was determined at 5 min with a video tracking system (Smart 3.0 tracking software, Panlab, Kent, UK).

Y-maze spontaneous alternation test, a measure of spatial working memory, was performed on the final day of behavioral testing as previously described.^22^ Briefly, the Y-maze apparatus comprised white Plexiglas (dimensions 38.5 × 8 × 13 cm, spaced 120° apart) and was illuminated with low lux (100 lux) lighting. Mice were placed in the maze and allowed to explore freely for 7 min, while tracking software recorded zone transitioning and locomotor activity (Smart 3.0 tracking software, Panlab, Kent, UK). Spontaneous alternation was calculated using the following formula: spontaneous alternation = 100 × number of alternations/(total arm entries − 2).

The novel object recognition (NOR), a measure of recognition memory, was performed as described previously.^22^ Briefly, on day 1, mice were habituated in a gray 50 × 50 × 50 cm apparatus illuminated with low lux (100 lux) lighting, and mice were placed into the empty maze and allowed to move freely for 10 min. On day 2, mice were conditioned to a single object for a 10 min period. On day 3, mice were placed into the same experimental area in the presence of two identical objects for 15 min, after which they were returned to their respective cages and an inter-trial interval of 1 h was observed. One familiar object was replaced with a novel object. Mice were placed back within the testing area for a final 10 min. Videos were analyzed for a 5 min period, after which if an accumulative total of 15 s with both objects failed to be reached, analysis continued for the full 10 min or until 15 s was achieved. Those not achieving 15 s were excluded from the analysis.^29^ A discrimination index (DI) was calculated as follows: DI = (TN−TF)/(TN+TF), where TN is the time spent exploring the novel object and TF is the time spent exploring the familiar object.

### Cell culture

The human cerebromicrovascular endothelial cell line hCMEC/D3 was maintained and treated as described previously.^30^ Cells were cultured to confluency in complete endothelial cell growth medium MV2 (PromoCell GmbH, Germany), whereupon VEGF was removed, and cells were further cultured for a minimum of 4 days to enable intercellular tight junction formation prior to experimentation. All cell cultures were used at passages 28–33 to ensure retention of appropriate endothelial characteristics, as is recommended for this cell line.^31^

### In vitro barrier function assessments

Paracellular permeability and TEER were measured on 100% confluent hCMEC/D3 cultures polarized by growth on 24-well plate polyethylene terephthalate (PET) transwell inserts (surface area: 0.33 cm^2^, pore size: 0.4 μm; Appleton Woods, UK) previously coated with calf-skin collagen (15 µg/cm^2^ and fibronectin 3 µg/cm^2^; both Sigma-Aldrich, UK). The permeability of hCMEC/D3 cell monolayers to 70 kDa FITC-dextran (2 mg/ml) was measured as described previously.^22^ TEER measurements were performed using a Millicell ERS-2 Voltohmmeter (Millipore, Watford, UK) and were expressed as Ω.cm^2^. In all cases, values obtained from cell-free inserts similarly coated with collagen and fibronectin were subtracted from the total values.

### Immunofluorescence analysis

hCMEC/D3 cells were cultured on Lab-Tek™ Permanox™ 8-well chamber slides coated with calf skin collagen (Sigma-Aldrich, UK), prior to immunostaining according to standard protocols^32^ and using a primary antibody directed against zona occludens-1 (ZO-1; 1:100, Thermo Fisher Scientific, UK) or AF488-conjugated phalloidin (0.165 µM, Thermofisher Scientific, UK). Nuclei were counterstained with DAPI (Sigma-Aldrich, UK). Images were captured using an LSM880 confocal laser scanning microscope (Carl Zeiss Ltd., Cambridge, UK) fitted with 405, 488, and 561 nm lasers and a × 63 oil immersion objective lens (NA, 1.4 mm, working distance, 0.17 mm). Images were captured with ZEN imaging software (Carl Zeiss Ltd., UK) and analyzed using ImageJ 1.53k (National Institutes of Health, USA).

### Flow cytometry

Following experimental treatment, hCMEC/D3 cells were detached from growth surfaces using 0.5% trypsin and fixed by incubation for 15 min at room temperature in 4% formaldehyde in 0.1 M PBS. Cells were permeabilised with ice-cold 90% methanol in 0.1 M PBS (10 min) and nonspecific secondary antibody binding was blocked with 10% fetal calf serum in 0.1 M PBS, 1 mm CaCl_2_ (FCS) for 20 min at 4°C. Primary antibodies used were as follows: ANXA1 (rabbit anti-human, 1:1000, ThermoFisher Scientific, UK), phosphotyrosine-21 ANXA1 (rabbit anti-human, 1:1000,^28^ EGFR (rabbit anti-human, 1:50, Cell Signalling Technologies, UK), phosphotyrosine-1068 EGFR (mouse anti-human, 1:1000, Cell Signalling Technologies, UK), MMP2 (rabbit anti-human, 1:500, Insight Biotechnology Ltd, UK), MMP9 (mouse anti-human, 1:50, Santa Cruz Biotechnology Inc., USA) or phosphotyrosine-705 STAT3 (mouse anti-human, 1:200, Cell Signalling Technologies, UK), all incubated with cells in 1% FCS for 20 min on ice. Cells were washed and incubated with either an AF488-conjugated goat anti-rabbit or an AF488-conjugated goat anti-mouse secondary antibody as appropriate (1:300, Thermo-Fisher Scientific, UK) in 1% FCS for 20 min on ice. Immunofluorescence was analyzed for 10,000 singlet events per treatment using a BD FACSCanto II (BD Biosciences, UK) flow cytometer, with resultant data being analyzed with FlowJo 8.0 software (Treestar Inc., CA, USA).

### Gelatinase activity analysis

Cell-free, serum-free, hCMEC/D3 conditioned medium was collected and concentrated 10-fold by centrifugal filtration (30 kDa cutoff, Pall Corporation, UK) at 14,000 *g* for 8 min, with resuspension in sterile 0.1 M PBS. Concentrated samples (10 µl/sample) were mixed with 5× non-reducing sample buffer (125 mm Tris. HCl, 4% sodium dodecyl sulfate (SDS), 20% glycerol, 0.01% bromophenol blue, pH 6.8; all Merck, UK) and separated by gel electrophoresis (7.5% acrylamide, 4 mg/ml porcine gelatin; Merck, UK). SDS was removed by washing in 50 mm Tris.HCl, 2.5% Triton X-100, 5 mm CaCl_2_, 1 µM ZnCl_2_, pH 7.5. Gelatinase activity was determined by incubation for 24 h in 50 mm Tris.HCl, 1% Triton X-100, 5 mm CaCl_2_, 1 µM ZnCl_2_, pH 7.5 followed by Coomassie staining (0.5% Coomassie brilliant blue, 40% methanol, 10% acetic acid). Gels were visualized using a ChemiDoc MP Imaging System (Bio-Rad Laboratories Ltd., UK). Integrated optical densities were calculated using NIH ImageJ 1.53k (National Institutes of Health, USA).

### EGFR siRNA

hCMEC/D3 cells grown on collagen/fibronectin-coated, 24-well plate PET transwell inserts (surface area: 0.33 cm^2^, pore size: 0.4 μm; Appleton Woods, UK) were transfected with one of three different commercial siRNA sequences designed to target the EGFR or an Allstars negative control siRNA sequence (final concentration 1 nM; all Qiagen Ltd., UK) using INTERFERin transfection reagent (Polyplus Transfection, France), alongside mock-transfected cells. After 96 h, cells were analyzed for TEER and paracellular 70 kDa FITC-dextran tracer permeability as described above. A proportion of cells were analyzed for EGFR expression by flow cytometry as described above (Supplemental Figure S1).

### ANXA1 shRNA

hCMEC/D3 cells bearing shRNA sequences targeting ANXA1 or a scramble sequence were used as described previously.^22^ Prior to use, reduction in ANXA1 expression was confirmed by flow cytometry as described above (Supplemental Figure S1).

### Cell survival analysis

The potential for pCS-induced cytotoxicity was assessed using the MTT assay. Briefly, cells were treated with pCS (10 µM, 100 µM, 1 mm) for 24 h, prior to administration of MTT (3-(4,5-dimethylthiazol-2-yl)-2,5-diphenyltetrazolium bromide; Merck Life Science UK Ltd., Gillingham, UK) at 500 μg/ml. Cells were incubated at 37°C for 2 h, medium was removed, and the resulting crystals were solubilized by incubation for 2 min in dimethyl sulfoxide. Absorbance was read at 540 nm using a CLARIOstar spectrophotometer (BMG Labtech, Ortenberg, Germany), with a reference wavelength at 570 nm.

### Reactive oxygen species production

Total intracellular ROS production was quantified using 6-chloromethyl-2‘,7’-dichlorodihydrofluorescein diacetate, acetyl ester (CM-H_2_DCFDA; Thermofisher Scientific, UK) as described previously.^33^ Following experimental treatment, fluorescence was determined every 5 minutes for 1 hr at 37°C using a CLARIOstar microplate reader (BMG Labtech, Germany) with excitation and emission filters set at 492 nm and 517 nm, respectively.

### LC-MS/MS

Serum samples were diluted with methanol at a ratio of 1:10 (v/v) and placed on dry ice for 10 min. Samples were then centrifuged (5 min 16,000 *g* at room temp), supernatants filtered using a 0.45 µm PTFE syringe filter and evaporated to dryness using a Savant™ SpeedVac™ High-Capacity Concentrator (Cat. SC210A–230). Dried samples were resuspended in 50 µl of water with 15 µl of L-tryptophan-2,3,3-d_3_ and *p*-toluenesulfonic acid at 50 µg/ml as the internal standards for tryptophan and *p*-cresol metabolites, respectively.

Stock solutions of each metabolite were prepared in methanol (1 mg/ml) and stored at −80°C. Calibration standards were prepared by pooling all analytes at eight different concentrations and adding the respective internal standards at 50 µg/ml. Calibration standards were run at the beginning, middle, and end of each analytical queue.

Two microliters of the prepared sample was injected and separated using a Waters Acquity UPLC system and Xevo TQ-S Cronos mass spectrometer and ACQUITY UPLC BEH C18 1.7 µm (2.1 × 50 mm) column. The electrospray ionization operated in positive mode and chromatic separation occurred using a 0.3 mL/min and composition of eluent A (0.1% formic acid, water) and eluent B (0.1% formic acid, methanol) at a gradient of 5% B from 0 to 0.5 min, 5%–10% B from 0.5 to 2.5 min, 10%–15% B from 2.5 to 3.5 min, 15%–35% B from 3.5 to 4.5 min, 35%–45% B from 4.5 to 6.5 min, 45%–55% B from 6.5 to 7 min, 55% = 100% B from 7 to 7.5 min, 100% B until 10 min, and 5% B from 10 to 10.1 min to return to initial conditions for equilibration until 14 min. The analyte:internal standard response ratio was used to create a calibration curve and quantify each metabolite.

### Statistical analyses

Sample sizes were calculated to detect differences of 15% or more with a power of 0.85 and α set at 5%, calculations being informed by previously published data.^22^ Experimental data are expressed as mean ± SEM, with *n* = 4–9 independent experiments for *in vitro* studies and *n* = 5–10 individual animals for *in vivo*/*ex vivo* studies (see figure legends for details of individual experiments). For *in vivo*/*ex vivo* studies, individual animals were randomly assigned to groups using the =rand() function of Microsoft Excel. Statistical analyses were performed using RStudio 2022.07.1. In all cases, normality of distribution was established using the Shapiro – Wilk test, followed by analysis with two-tailed Student’s *t*-tests to compare two groups or, for multiple comparisons, one- or two-way ANOVA with Tukey’s HSD *post hoc* test as appropriate. A *p* value of <5% was considered significant.

## Results

### Treatment with pCS increases brain endothelial permeability in vitro and in vivo

**Figure 1.**
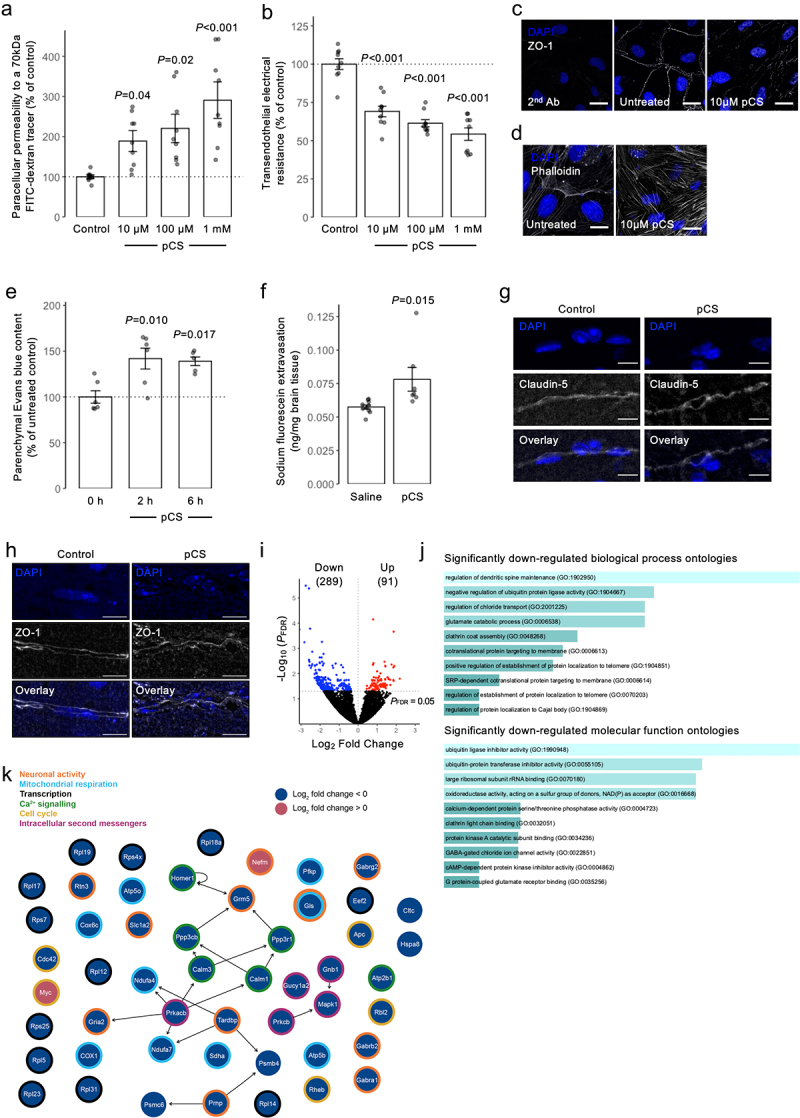
Exposure to pCS increases the permeability of human cerebromicrovascular endothelial cells *in vitro* and the murine BBB *in vivo*. (a) Incubation of hCMEC/D3 cell monolayers with pCS (10 µm, 100 µm, 1 mm; 24 h) dose-dependently increased paracellular permeability to a 70 kDa fitc-dextran conjugate; data are mean ± s.e.m., *n* = 9 independent experiments. (b) incubation of hCMEC/D3 cell monolayers with pCS (10 µm, 100 µm, 1 mm; 24 h) dose-dependently reduced TEER; data are mean ± s.e.m., *n* = 9 independent experiments. (c, d) confocal microscopic analysis of expression of C) AF488-phalloidin labeled actin filaments (white) or d) the tight junction component zona occludens-1 (ZO-1; white) in hCMEC/D3 cells following treatment for 24 h with 10 µm pCS, nuclei are counterstained with DAPI (blue), scale = 15 µm. Images are representative of at least three independent experiments. (e) treatment of male C57Bl/6 mice by i.P. injection of pCS (10 mg/kg) caused a time-dependent increase in extravasation of Evans blue tracer into the CNS parenchyma, reaching statistical significance at both 2 h and 6 h post administration; data are mean ± s.e.m., *n* = 5-6 animals. (f) exposure of male C57Bl/6 by s.C. implantation with osmotic mini-pumps releasing pCS at 7.5 µg/h for 28 days significantly enhanced extravasation of sodium fluorescein into the brain parenchyma; data are mean ± s.e.m., *n* = 7-10 animals. (g, h) confocal microscopic analysis of expression of G) claudin-5 or H) zonula occludens-1 (ZO-1) in the brains of male C57Bl/6 exposed by s.C. implantation with osmotic mini-pumps releasing pCS at 7.5 µg/h for 28 days, nuclei are counterstained with DAPI (blue), scale = 10 µm. Images are representative of 7-10 animals; (i) volcano plot showing the 380 significantly differentially expressed genes among the 16,988 mouse genes examined in this study (*n* = 5 animals per group (treated, negative control)). Black data points: no significant change in expression; red dots, significantly (*p* < 0.05, Benjamini-Hochberg) upregulated expression in the pCS group compared with the negative control; blue dots, significantly (*p* < 0.05, Benjamini-Hochberg) downregulated expression in the pCS group compared with the negative control; *n* = 5 per group. (j) biological processes (above) and molecular functions (below) of genes found to be significantly downregulated (*n* = 289) upon exposure of mice to pCS, based on enrichr *p* value ranking from gene ontology analysis. (k) topological analysis of the KEGG network resulting from mapping the 380 significantly (*p* < 0.05, Benjamini-Hochberg) differentially expressed genes onto the 17 KEGG *Mus musculus* metabolic pathways listed in Table 1. Blue solid circles, genes whose expression is significantly downregulated in the pCS group compared with the control; red solid circles, genes whose expression is significantly upregulated in the pCS group compared with the control. Thick outline colors around the solid circles correspond to the molecular functions listed in the legend. Network summary statistics are available in table S3.

To assess the effects of pCS upon BBB integrity, we first examined its effects upon a simple *in vitro* model of BBB function, namely the paracellular permeability barrier of a monolayer of the immortalized human cerebromicrovascular endothelial line hCMEC/D3.^31^ Treatment with pCS (24 h; 10 µM, 100 µM, or 1 mm) significantly (*p* < 0.05) and dose-dependently enhanced permeability to a 70 kDa FITC-dextran tracer (Figure 1a) and reduced trans-endothelial electrical resistance (TEER; Figure 1b). Notably, pCS showed no signs of endothelial toxicity at any concentration tested (Supplemental Figure S2), and given that pCS was administered as a potassium salt, equivalent concentrations of KCl had no effect on barrier function (Supplemental Figure S2). Moreover, chelation of pCS by inclusion of 50 mg/ml human albumin (approximately equivalent to human serum concentrations^34^) blocked its effects on paracellular permeability and TEER (Supplemental Figure S3). The BBB permeability barrier is underpinned by tight inter-endothelial junctions and their interactions with the actin cytoskeleton; hence, we examined the effect of pCS treatment upon the key tight junction component zonula occludens-1 (ZO-1) and fibrillar actin in hCMEC/D3 monolayers. In comparison with untreated cells, pCS exposure (24 h, 10 µM) markedly disrupted the marginal localization of ZO-1 (Figure 1c), as well as disrupting actin fibril arrangement itself (Figure 1d), with a clear loss of cortical actin and appearance of trans-cytoplasmic fibers.

To confirm that these *in vitro* findings had relevance for whole animal physiology, we exposed male C57Bl/6 mice to pCS (10 mg/kg i.p., chosen to approximately double circulating free pCS concentrations^21^; 2 h, 6 h) before assessing BBB permeability through cerebral extravasation of the albumin-binding tracer Evans blue. Treatment of mice with pCS resulted in a significant (*p* < 0.02) increase in brain parenchymal Evans blue at both 2 h and 6 h post-administration (Figure 1e).

CKD is characterized in part by persistently elevated circulating levels of pCS; hence, we examined how continuous pCS release from implanted osmotic minipumps for four weeks affected the BBB, neuroinflammation, and cognitive function (see Supplemental Table S2 for serum metabolite content analysis). Animals so exposed to pCS exhibited both significantly enhanced extravasation of the tracer sodium fluorescein into the brain parenchyma (Figure 1f) and disrupted expression of the key BBB inter-endothelial tight junction molecules claudin-5 (Figure 1g) and ZO-1 (Figure 1h), confirming the deleterious effects seen with acute pCS administration. However, no obvious sign of microglial or astrocytic change was seen in the cortex of these animals (Supplemental Figure S4), nor was performance in the open field test, Y-maze or novel object recognition task significantly affected (Supplemental Figure S5) in comparison to saline-treated animals.

### Murine whole brain transcriptomic analysis reveals a suppressive effect of pCS exposure upon neuronal activity

To investigate potential mechanisms of pCS action, we performed RNAseq analysis of whole-brain transcriptomes, comparing untreated and pCS-exposed mice (10 mg/kg i.p., 2 h). We found 380/16,988 genes assayed were significantly differentially expressed (*p* < 0.05, Benjamini-Hochberg): 91 genes upregulated and 289 downregulated in the pCS-exposed group (Figure 1i; Supplemental Table S3). Among BBB-specific genes, the expression of *Slc1a2* (log_2_ fold change −1.35), *Adgra2* (1.03) and *Serpini1* (−1.93) was found to be significantly changed (*p* < 2.66 × 10^−4^ for all, Benjamini-Hochberg).

**Figure 2.**
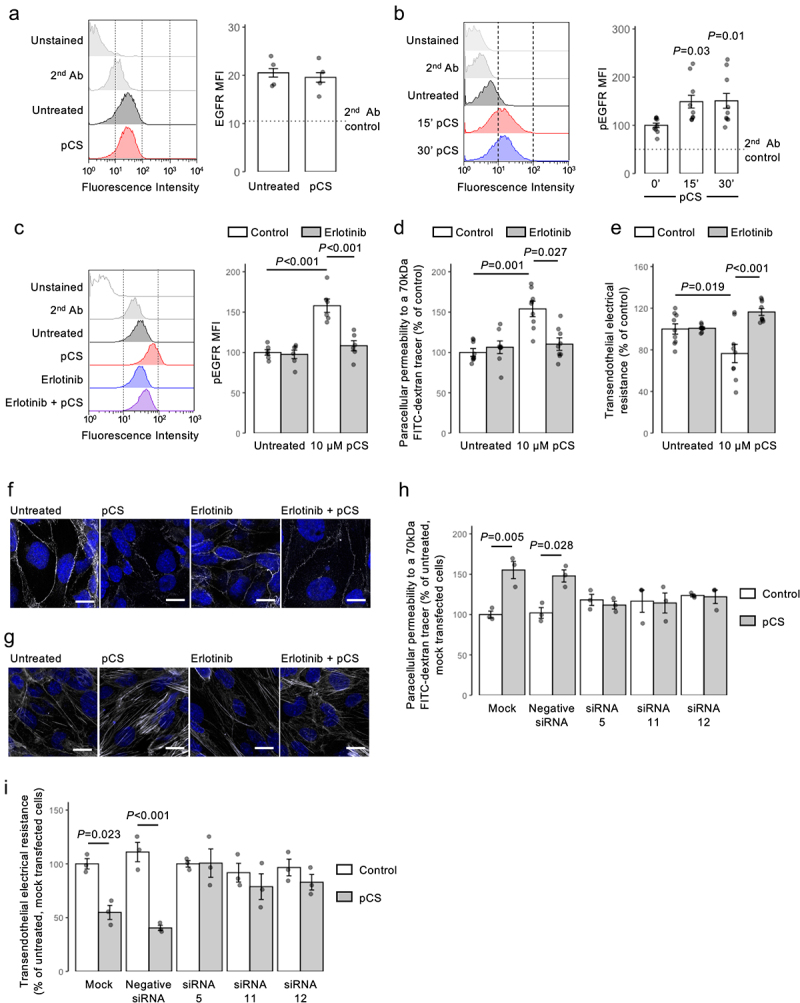
pCS regulates cerebromicrovascular cell permeability *in vitro* through activation of the EGFR. (a) Stimulation of hCMEC/D3 cells with pCS (24 h, 10 µm) has no effect on cell surface expression of EGFR; data are mean ± s.e.m., *n* = 4 independent experiments; representative flow cytometry histograms are shown. (b) treatment of hCMEC/D3 cells with pCS (10 µm) causes a time-dependent increase in EGFR Tyr1068 phosphorylation, maintained for at least 30 min; data are mean ± s.e.m., *n* = 9 independent experiments; representative flow cytometry histograms are shown. (c) pre-treatment of hCMEC/D3 cells with erlotinib (2.5 µm, 10 min) prevents EGFR Tyr1068 phosphorylation in response to pCS treatment (10 µm, 30 min), data are mean ± s.e.m., *n* = 6 independent experiments; representative flow cytometry histograms are shown. (d) pre-treatment of hCMEC/D3 cell monolayers with erlotinib (2.5 µm, 10 min) prevents the increase in paracellular permeability to a 70 kDa fitc-dextran conjugate induced by pCS stimulation (10 µm, 24 h); data are mean ± s.e.m., *n* = 9 independent experiments. (e) pre-treatment of hCMEC/D3 cell monolayers with erlotinib (2.5 µm, 10 min) prevents the reduction in TEER induced by pCS stimulation (10 µm, 24 h); data are mean ± s.e.m., *n* = 9 independent experiments. (f) confocal microscopic analysis of expression of the tight junction component zona occludens-1 (ZO-1; white) in hCMEC/D3 cells following treatment with either pCS (10 µm, 24 (h), erlotinib (2.5 µm, 24 h) or both (erlotinib 2.5 µm, 10 min pre-treatment, followed by pCS 10 µm, 24 h); nuclei are counterstained with DAPI (blue), scale bar = 15 µm. Images are representative of at least three independent experiments. (g) confocal microscopic analysis of expression of AF488-conjugated phalloidin labeled actin filaments (white) in hCMEC/D3 cells following treatment with either pCS (10 µm, 24 h), erlotinib (2.5 µm, 24 h) or both (erlotinib 2.5 µm, 10 min pre-treatment, followed by pCS 10 µm, 24 h); nuclei are counterstained with DAPI (blue), scale bar = 15 µm. Images are representative of at least three independent experiments. (h) paracellular permeability to a 70 kDa fitc-dextran conjugate of hCMEC/D3 cell monolayers bearing siRNA sequences targeting the EGFR (siRNA 5, siRNA 11, siRNA 12), a non-targeting negative control siRNA sequence, or that had been mock-transfected, with or without stimulation with pCS (10 µm, 24 h); data are mean ± s.e.m., *n* = 4 independent experiments. (i) TEER of hCMEC/D3 cell monolayers bearing siRNA sequences targeting the EGFR (siRNA 5, siRNA 11, siRNA 12), a non-targeting negative control siRNA sequence, or that had been mock-transfected, with or without stimulation with pCS (10 µm, 24 h); data are mean ± s.e.m., *n* = 3 independent experiments.

Analysis of over-represented Gene Ontology categories within differentially expressed genes using Enrichr^24^ revealed that, while no categories associated with significantly up-regulated genes reached statistical significance after multiple testing correction, several biological processes and molecular functions associated with significantly down-regulated genes could be identified (Figure 1j). Broadly, these fell into two main categories: ontologies reflective of neuronal function and synaptic activity and those indicating intracellular protein translocation and metabolism. Signalling Pathway Impact Analysis (SPIA) demonstrated inhibition of pathways associated with glutamatergic synapse, circadian entrainment, and Alzheimer’s disease (pGFWER < 0.05; Table 2). Topological analysis of the network generated from the 380 significantly differentially expressed genes mapped onto KEGG pathways identified in SPIA (pGFdr < 0.05; Table 2) suggested that the genes for *Prkacb*, *Homer1*, *Tardbp*, *Calm1*, *Calm3*, *Ppp3r1*, *Ppp3cb*, and *Grm5* had most control over the network (Figure 1k; Supplemental Table S4). Manual curation of SPIA-highlighted genes identified several categories: neuronal activity, mitochondrial respiration, translation, cell cycle activity, and intracellular Ca^2+^- and other signaling pathways (Figure 2c), overall depicting a generalized inhibitory effect of pCS upon brain function.

**Table 2.** Significant results generated from signalling pathway impact analysis for 380 significantly differentially expressed genes. pGfdr: Benjamini–Hochberg correction (false discovery rate), pGFWER: Bonferroni correction (family-wise error rate).

Name	KEGG pathway ID	pGFdr	pGFWER	Status
Glutamatergic synapse	mmu:04724	2.83 × 10^−4^	2.83 × 10^−4^	Inhibited
Circadian entrainment	mmu:04713	2.98 × 10^−3^	5.97 × 10^−3^	Inhibited
Alzheimer disease	mmu:05010	1.40 × 10^−2^	4.20 × 10^−2^	Inhibited
Amphetamine addiction	mmu:05031	1.92 × 10^−2^	7.68 × 10^−2^	Inhibited
Oxytocin signaling pathway	mmu:04921	2.54 × 10^−2^	1.27 × 10^−1^	Inhibited
Glucagon Signalling pathway	mmu:04922	2.75 × 10^−2^	1.72 × 10^−1^	Inhibited
Retrograde endocannabinoid signaling	mmu:04723	2.75 × 10^−2^	1.92 × 10^−1^	Inhibited
Pathways of neurodegeneration – multiple diseases	mmu:05022	2.98 × 10^−2^	2.66 × 10^−1^	Inhibited
VEGF signaling pathway	mmu:04370	2.98 × 10^−2^	2.68 × 10^−1^	Inhibited
Cellular senescence	mmu:04218	3.12 × 10^−2^	3.13 × 10^−1^	Inhibited
Aldosterone synthesis and secretion	mmu:04925	3.12 × 10^−2^	3.43 × 10^−1^	Inhibited
Ras signaling pathway	mmu:04014	4.14 × 10^−2^	4.96 × 10^−1^	Inhibited
C-type lectin receptor signaling pathway	mmu:04625	4.18 × 10^−2^	5.44 × 10^−1^	Inhibited
Coronavirus disease – COVID-19	mmu:05171	4.59 × 10^−2^	6.42 × 10^−1^	Activated
Lipid and atherosclerosis	mmu:05417	4.83 × 10^−2^	7.24 × 10^−1^	Inhibited
Gastric acid secretion	mmu:04971	4.94 × 10^−2^	7.90 × 10^−1^	Inhibited
Huntington disease	mmu:05016	4.97 × 10^−2^	8.45 × 10^−1^	Inhibited

### Permeabilizing effects of pCS are mediated through the EGFR

Although transcriptomic analysis did not suggest a clear candidate pathway for the actions of pCS, a previous study has indicated an interaction with the EGFR, albeit at supra-physiological free concentrations and in an epithelial cell line,^35^ hence we investigated whether EGFR might mediate the effects of pCS upon the BBB. Initial studies of hCMEC/D3 cells confirmed EGFR expression (Figure 2a) and rapid pCS-dependent Tyr1068 phosphorylation (Figure 2b), a signaling response sensitive to the EGFR-specific inhibitor erlotinib (2.5 µM, 10 min pre-treatment; Figure 2c). Additionally, as pCS has been reported to cause oxidative stress,^19,36^ we examined whether this held true for hCMEC/D3 cells. However, while exposure to 1 mm pCS did indeed induce reactive oxygen species production within 1 h of exposure, this was not the case for lower concentrations of pCS tested (1 µM, 10 µM, 100 µM). As free pCS in the serum of patients with CKD is generally toward the lower end of this range,^37^ we considered this route of action unlikely to play a major role in the effects on the BBB we describe (Supplemental Figure S6).

Considering these interactions, we investigated whether EGFR activation mediated the permeabilizing actions of pCS upon hCMEC/D3 cells. Erlotinib pre-treatment (2.5 µM, 10 min pre-treatment) efficiently ablated the pCS-induced (10 µM, 24 h) disruption to paracellular permeability (Figure 2d) and TEER (Figure 2e). These functional effects were paralleled by a reduction in pCS-stimulated disruption to the intracellular distribution of the tight junction molecule ZO-1 (Figure 2f) and to the actin cytoskeleton (Figure 2g).

While erlotinib has high selectivity for the EGFR, it is not without off-target effects; hence, we confirmed the centrality of this receptor in pCS action through use of targeted siRNA-mediated knockdown. Treatment of hCMEC/D3 cells expressing any of three independent siRNA sequences (knockdown efficiencies of 65.3%, 56.7%, and 76.8%, respectively, Supplemental Fig. S1A) with pCS (10 µM, 24 h) had little effect on either paracellular permeability (Figure 2h) or TEER (Figure 2i). This was in marked contrast to cells that had been either mock-transfected or bearing non-targeting siRNA sequences, in which pCS exposure significantly (*p* < 0.05) increased paracellular permeability (Figure 2h) and significantly (*p* < 0.05) decreased TEER (Figure 2i), closely aligning with previous experiments.

### pCS stimulation of EGFR activates an ANXA1–STAT3–MMP signalling pathway

**Figure 3.**
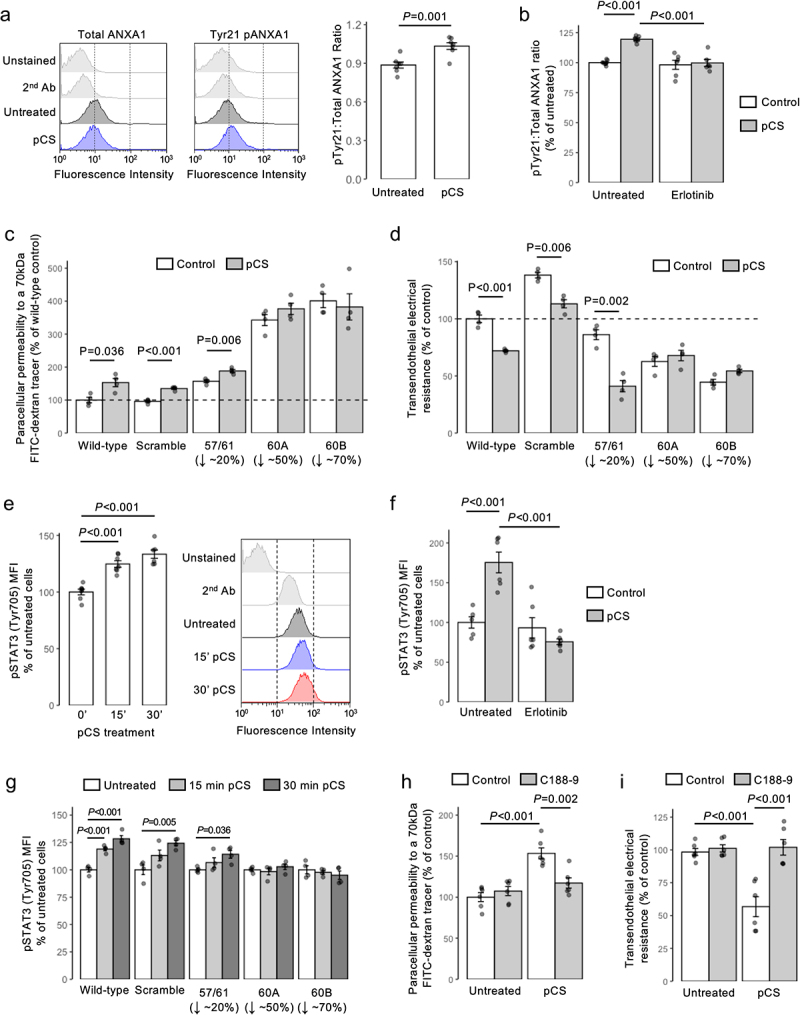
Stimulation of EGFR by pCS activates a downstream ANXA1–STAT3 signaling pathway. (a) Stimulation of hCMEC/D3 cells with pCS (10 µm, 30 min) increases the ratio of Tyr21 phosphorylated to total annexin A1; data are mean ± s.e.m., *n* = 7 independent experiments; representative flow cytometry histograms are shown. (b) pre-treatment of hCMEC/D3 cells with erlotinib (2.5 µm, 10 min) prevents the increase in the Tyr21 phosphorylated to total ANXA1 expression ratio induced by pCS stimulation (10 µm, 30 min); data are mean ± s.e.m., *n* = 6 independent experiments. (c) paracellular permeability to a 70 kDa fitc-dextran tracer of monolayers of wild-type hCMEC/D3 cells, or hCMEC/D3 cells stably transfected with either a scramble shRNA sequence or one of three independent shRNA sequences targeting ANXA1 following stimulation with pCS (10 µm, 24 h); data are mean ± s.e.m., *n* = 4 independent experiments. (d) TEER of monolayers of wild-type hCMEC/D3 cells, or hCMEC/D3 cells stably transfected with either a scramble shRNA sequence or one of three independent shRNA sequences targeting ANXA1 following stimulation with pCS (10 µm, 24 h); data are mean ± s.e.m., *n* = 4 independent experiments. (e) stimulation of hCMEC/D3 cells with pCS (10 µm) causes a time-dependent increase in expression of Tyr705 phosphorylated STAT3; data are mean ± s.e.m., *n* = 7 independent experiments; representative flow cytometry histograms are shown. (f) pre-treatment of hCMEC/D3 cells with erlotinib (2.5 µm, 10 min) prevents the increase in Tyr705 phosphorylated STAT3 expression induced by pCS treatment (10 µm, 30 min); data are mean ± s.e.m., *n* = 6 independent experiments. (g) expression of Tyr705 phosphorylated STAT3 in wild-type hCMEC/D3 cells, or hCMEC/D3 cells stably transfected with either a scramble shRNA sequence or one of three independent shRNA sequences targeting ANXA1 following stimulation with pCS (10 µm), expressed as percentage of untreated cells; data are mean ± s.e.m., *n* = 4 independent experiments. (h) pre-treatment of hCMEC/D3 cell monolayers with the STAT3 inhibitor C188-9 (5 µm, 30 min) prevents the increase in paracellular permeability to a 70 kDa fitc-dextran conjugate induced by pCS stimulation (10 µm, 24 h); data are mean ± s.e.m., *n* = 6 independent experiments. (i) pre-treatment of hCMEC/D3 cell monolayers with C188-9 (5 µm, 30 min) prevents the reduction in TEER induced by pCS stimulation (10 µm, 24 h); data are mean ± s.e.m., *n* = 6 independent experiments.

EGFR is coupled to a number of intracellular signaling pathways, but one potentially relevant target is the protein annexin A1 (ANXA1),^38^ a molecule we have previously shown to regulate BBB function.^39^ Initial experiments confirmed that ANXA1 underwent Tyr21 phosphorylation in response to pCS exposure (10 µM, 30 min; Figure 3a) in an erlotinib-sensitive manner (2.5 µM, 10 min pre-treatment; Figure 3b). Using hCMEC/D3 cells stably bearing shRNA sequences to ANXA1^22^ (Supplemental Figure S1B), we further identified that pCS (10 µM, 24 h) significantly enhanced paracellular permeability in monolayers of wild-type and scramble shRNA-bearing hCMEC/D3 cells, but that this response was absent in cells transfected with ANXA1 shRNA sequences (Figure 3c). Similarly, TEER was significantly (*p* < 0.01) reduced in wild-type and scramble shRNA-bearing hCMEC/D3 cells upon pCS exposure, but not in ANXA1 shRNA transfected cells (Figure 3d).

Both EGFR signaling and ANXA1 Tyr21 phosphorylation have been linked with the transcription factor STAT3,^40,41^ hence we examined the impact of pCS exposure upon STAT3 activation. Exposure of hCMEC/D3 cells to pCS (10 µM) significantly (*p* < 0.001) enhanced STAT3 Tyr705 phosphorylation within 15 min (Figure 3e), an effect that was not seen in cells pre-treated with erlotinib (2.5 µM, 10 min pre-treatment; Figure 3f) and was attenuated in hCMEC/D3 cells bearing ANXA1 shRNA sequences (Figure 3g). Notably, pre-treatment of hCMEC/D3 cells with the selective STAT3 inhibitor C188–9 (5 µM, 30 min pre-treatment) significantly (*p* < 0.002) reduced the effects of pCS exposure (10 µM, 24 h) upon both paracellular permeability (Figure 3h) and TEER (Figure 3i).

### Inhibition of MMP2/9 activity prevents the permeabilizing effects of pCS in vitro

**Figure 4.**
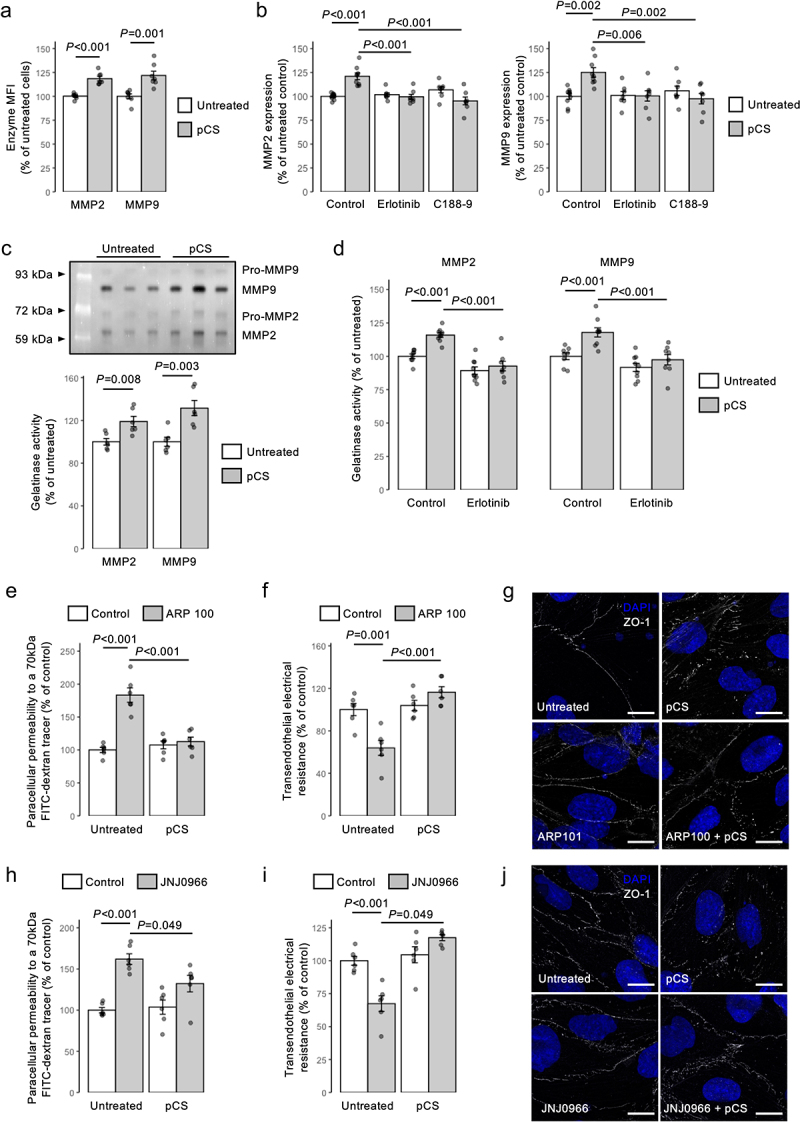
pCS enhances endothelial permeability *via* mobilization of active MMP2 and MMP9. (a) Treatment of hCMEC/D3 cells with pCS (10 µm, 24 h) increases expression of MMP2 and MMP9; data are mean ± s.e.m., *n* = 7 independent experiments. (b) stimulation of MMP2 or MMP9 expression in hCMEC/D3 cells by pCS (10 µm, 24 h) is prevented by either erlotinib (2.5 µm, 10 min pre-treatment) or C188-9 (5 µm, 30 min pre-treatment); data are mean ± s.e.m., *n* = 6 independent experiments. (c) active MMP2 and MMP9 are secreted into the culture medium of hCMEC/D3 cells in response to pCS (10 µm, 24 h) stimulation; data are mean ± s.e.m., *n* = 6 independent experiments; a typical gelatin zymography is shown, highlighting both active MMP2 and MMP9 and artifactually revealed pro-MMP2 and pro-MMP9 activity. (d) active MMP2 and MMP9 release from hCMEC/D3 cells into culture medium in response to pCS (10 µm, 24 h) stimulation is prevented by erlotinib (2.5 µm, 10 min pre-treatment); data are mean ± s.e.m., *n* = 11-12 independent replicates. e) pre-treatment of hCMEC/D3 cell monolayers with the MMP2 inhibitor ARP 100 (12 nM, 15 min) prevents the increase in paracellular permeability to a 70 kDa fitc-dextran conjugate induced by pCS stimulation (10 µm, 24 (h); data are mean ± s.e.m., *n* = 6 independent experiments. (f) pre-treatment of hCMEC/D3 cell monolayers with ARP 100 (12 nM, 15 min) prevents the reduction in TEER induced by pCS stimulation (10 µm, 24 h); data are mean ± s.e.m., *n* = 6 independent experiments. (g) confocal microscopic analysis of expression of the tight junction component zona occludens-1 (ZO-1; white) in hCMEC/D3 cells following treatment with either pCS (10 µm, 24 h), ARP 100 (12 nM, 24 h) or both (ARP 100 12 nM, 15 min pre-treatment, followed by pCS 10 µm, 24 h); nuclei are counterstained with DAPI (blue), scale = 15 µm. Images are representative of at least three independent experiments. (h) pre-treatment of hCMEC/D3 cell monolayers with the MMP9 inhibitor JNJ0966 (440 nM, 15 min) prevents the increase in paracellular permeability to a 70 kDa fitc-dextran conjugate induced by pCS stimulation (10 µm, 24 h); data are mean ± s.e.m., *n* = 6 independent experiments. (i) pre-treatment of hCMEC/D3 cell monolayers with JNJ0966 (440 nM, 15 min) prevents the reduction in TEER induced by pCS stimulation (10 µm, 24 h); data are mean ± s.e.m., *n* = 6 independent experiments. (j) confocal microscopic analysis of expression of the tight junction component zona occludens-1 (ZO-1; white) in hCMEC/D3 cells following treatment with either pCS (10 µm, 24 h), JNJ0966 (440 nM, 24 h) or both (JNJ0966 440 nM, 15 min pre-treatment, followed by pCS 10 µm, 24 h); nuclei are counterstained with DAPI (blue), scale = 15 µm. Images are representative of at least three independent experiments.

Activation of MMPs, particularly that of MMP-2 and MMP-9, is known to be downstream of EGFR^35^ and STAT3^42^ activation, to be capable of damaging BBB permeability,^43^ and to be associated with CKD,^44^ leading us to hypothesize that the permeabilizing effects of pCS are mediated through these enzymes. Stimulation of hCMEC/D3 cells with pCS (10 µM, 24 h) significantly (*p* < 0.001) increased MMP-2 and MMP-9 expression (Figure 4a), a response inhibited by pre-treatment with either the EGFR inhibitor erlotinib (2.5 µM, 10 min pre-treatment) or the STAT3 inhibitor C188–9 (5 µM, 30 min pre-treatment; Figure 4b). Analysis of hCMEC/D3 conditioned medium by gelatin zymography confirmed that these pCS-induced (10 µM, 24 h) increases in cellular MMP-2 and MMP-9 expression were mirrored by enhanced gelatinase activity (Figure 4c), an increase again sensitive to erlotinib pre-treatment (2.5 µM, 30 min prior to 10 µM pCS; Figure 4d).

To judge the relevance of this increase in MMP activity to endothelial barrier integrity, we investigated the effect of selective MMP inhibitors upon the actions of pCS. Pre-treatment of hCMEC/D3 monolayers with either the selective MMP-2 inhibitor ARP 100 (12 nM, 15 min pre-treatment) or the selective MMP-9 inhibitor JNJ0966 (440 nM, 15 min pre-treatment) prevented the disruptive effects of pCS exposure (10 µM, 24 h) upon paracellular permeability to a 70 kDa FITC-dextran tracer (Figure 4e,h), TEER (Figure 4f,i) and intracellular distribution of the tight junction anchor ZO-1 (Figure 4g,j).

### pCS impairs BBB integrity through an EGFR–STAT3–MMP-9 pathway in vivo

**Figure 5.**
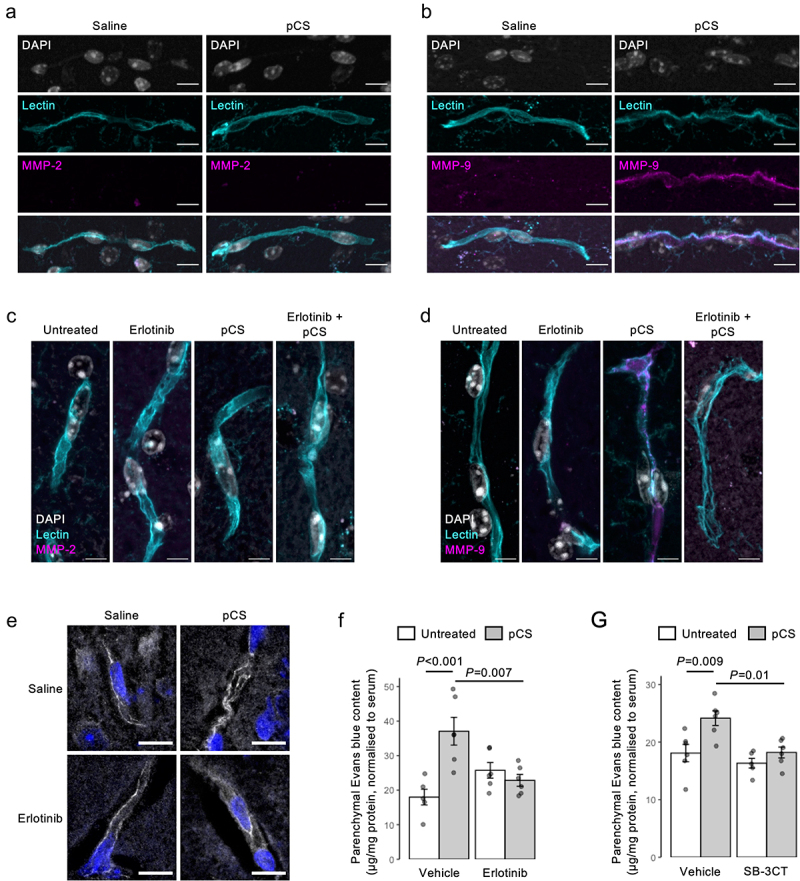
Treatment with pCS induces BBB permeability *in vivo* throughEGFR-dependent mobilization of MMP9. a, b) Confocal microscopic analysis of expression of (a) MMP-2 or (b) MMP-9 (magenta) in the brains of male C57Bl/6 exposed to pCS by s.C. implantation with osmotic mini-pumps releasing at 7.5 µg/h for 28 days, blood vessels are defined by tomato lectin binding (cyan), nuclei are counterstained with DAPI (white), scale = 10 µm, images are representative of 7-10 animals. c, d) confocal microscopic analysis of (c) MMP2 and (d) MMP9 expression (magenta) within the cerebral microvasculature of male C57Bl/6 mice exposed to pCS (10 mg/kg, i.P. 2 h) with or without erlotinib pre-treatment (50 mg/kg, i.P. 1 h pre-treatment); blood vessels are defined by tomato lectin binding (cyan), nuclei are counterstained with DAPI (white), scale bar = 5 µm, images are representative of 7-10 animals. (e) typical expression of ZO-1 (white) within the cerebral microvasculature of male C57Bl/6 mice exposed to pCS (10 mg/kg, i.P. 2 h) with or without erlotinib pre-treatment (50 mg/kg, i.P. 1 h pre-treatment); nuclei are counterstained with DAPI (blue), scale bar = 10 µm. (f) Increased extravasation of Evans blue tracer into the parenchyma of male C57Bl/6 mice following i.p. injection of pCS (10 mg/kg, 2 h exposure) is prevented by erlotinib administration (50 mg/kg i.p., 1 h pre-treatment); data are mean ± s.e.m., *n* = 5–6 animals. (g) Increased extravasation of Evans blue tracer into the parenchyma of male C57Bl/6 mice following i.p. injection of pCS (10 mg/kg, 2 h exposure) is prevented by administration of the dual-specific MMP2/MMP9 inhibitor SB-3CT (10 mg/kg i.p., 1 h pre-treatment); data are mean ± s.e.m., *n* = 5–6 animals.

We sought to validate the pCS-EGFR-MMP-2/9 pathway as damaging the BBB *in vivo*. Exposure of male C57Bl/6 mice to elevated circulating pCS for 4 weeks (~30 µM, see Supplemental Table S2) clearly increased brain blood vessel-associated MMP-9 (but not MMP-2) expression (Figure 5a–b), confirming the pCS-MMP link. Furthermore, pre-treatment of male C57Bl/6 mice with the EGFR inhibitor erlotinib (50 mg/kg, i.p., 1 h pre-treatment) prior to pCS injection (10 mg/kg i.p., 2 h) suppressed the pCS-induced appearance of Tyr21 phospho-ANXA1 (Supplemental Fig. S7) and MMP-9 (Figure 5c–d) within the cerebrovascular wall, ameliorated pCS-induced disruption of vascular ZO-1 expression (Figure 5e), and prevented a pCS-induced increase in parenchymal Evans blue tracer extravasation (Figure 5f). Confirming the importance of pCS-induced MMP activity in mediating BBB disruption, pre-treatment of mice with the MMP-2/9 dual-specific inhibitor SB-3CT (10 mg/kg i.p., 1 h pre-treatment) also prevented pCS treatment (10 mg/kg i.p., 2 h) from increasing parenchymal Evans blue extravasation (Figure 5g).

### Treatment with the EGFR inhibitor erlotinib can prevent the permeabilizing effect of CKD patient serum upon hCMEC/D3 monolayers

Kidney failure is associated with changes in the circulating levels of numerous uremic retention molecules, of which pCS is only one, albeit important, example. Most notably, the EGFR has been demonstrated to interact with the gut microbe-derived uremic toxin indoxyl sulfate (IS),^45^ although treatment of hCMEC/D3 monolayers with this metabolite was without effect on paracellular permeability or TEER at any concentration tested (5 µM, 25 µM, 50 µM, 150 µM; Supplemental Figure S8).

**Figure 6.**
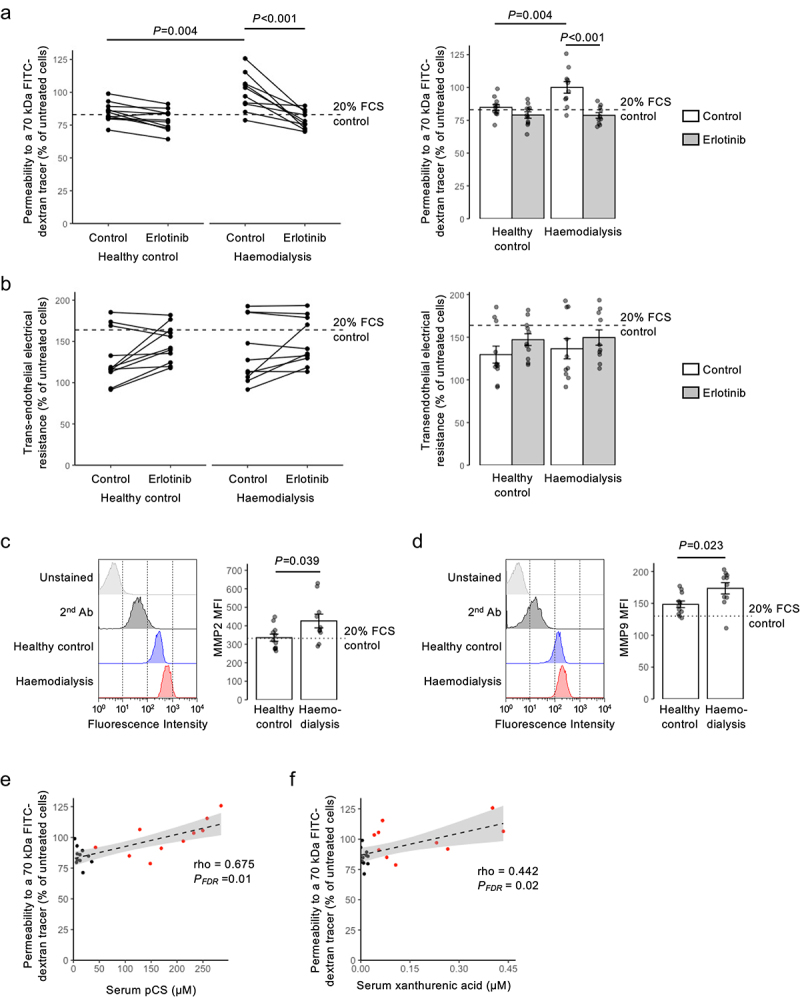
Sera from human hemodialysis patients significantly enhances endothelial permeability, an effect prevented by erlotinib pre-treatment. (a) Treatment of hCMEC/D3 monolayers with medium containing de-complemented sera from patients undergoing hemodialysis (20%, 24 h) significantly enhances permeability to a 70 kDa fitc-dextran tracer, an effect not seen with equivalent sera from healthy donors, and prevented by inclusion of erlotinib (2.5 µm, 10 min); data are mean ± s.e.m., *n* = 10-11 donors. (b) TEER of hCMEC/D3 monolayers is not affected by treatment with media containing sera (20%, 24 h) from either hemodialysis patients or healthy donors, nor by erlotinib inclusion (2.5 µm, 10 min pre-treatment); data are mean ± s.e.m., *n* = 10-11 donors. (c) treatment of hCMEC/D3 cells with medium containing de-complemented sera (20%, 24 h) from patients undergoing hemodialysis, but not with that from healthy donors, increases expression of MMP2; data are mean ± s.e.m., *n* = 10-11 donors. (d) treatment of hCMEC/D3 cells with medium containing de-complemented sera (20%, 24 h) from patients undergoing hemodialysis, but not with that from healthy donors, increases expression of MMP9; data are mean ± s.e.m., *n* = 10-11 donors. (e) correlation between concentrations of pCS in human sera samples (healthy donors in black, hemodialysis patients in red) with 70 kDa fitc-dextran permeability data from (A) above; *n* = 10-11 donors. (f) correlation between concentrations of xanthurenic acid in human sera samples (healthy donors in black, hemodialysis patients in red) with 70 kDa fitc-dextran permeability data from (A) above; *n* = 10-11 donors.

To investigate the relative importance of pCS in mediating the cerebrovascular symptoms of CKD, we exposed hCMEC/D3 monolayers for 24 h to medium containing 20% de-complemented serum from individuals undergoing hemodialysis or age-matched individuals with normal kidney function (Table 1), either alone or in combination with erlotinib (2.5 µM, 10 min pre-treatment). Sera from hemodialysis patients, but not that of healthy donors, significantly (*p* = 0.004) enhanced paracellular permeability, an effect reversed by erlotinib pre-treatment (Figure 6a), although notably none of these treatments affected TEER (Figure 6b). Moreover, treatment of hCMEC/D3 cells with sera from hemodialysis patients, but again not with that of healthy donors, significantly (*p* < 0.05) increased expression of both MMP-2 (Figure 6c) and MMP-9 (Figure 6d).

We examined the relative contributions of different amino acid-derived 7Following analysis of serum concentrations of different metabolites by LC-MS/MS (Table 3; mean pCS concentrations: healthy donor 14.12 ± 3.09 µM, hemodialysis patient 155.26 ± 27.36 µM, *p* < 0.001), we correlated these with changes in paracellular permeability; only correlations with pCS (rho = 0.675, *P*_*FDR*_ = 0.01; Figure 6e) and the tryptophan metabolite xanthurenic acid (rho = 0.442, *P*_*FDR*_ = 0.02; Figure 6f) remained significant following correction for multiple testing. Analysis of the response of hCMEC/D3 cells to xanthurenic acid revealed that while this molecule could slightly, but significantly, affect permeability to a 70 kDa tracer, this was without concurrent effects on TEER, nor were its effects sensitive to erlotinib pre-treatment (Supplemental Figure S9), suggesting the primacy of pCS amongst measured uremic toxins as modulating BBB function.

**Table 3.** Human hemodialysis patient serum metabolite concentrations. LC-MS/MS determined concentrations of different potential uremic toxins in sera from healthy donors and individuals undergoing hemodialysis for nephropathy, alongside coefficients of correlation between metabolite concentration and permeability of hCMEC/D3 cells to a 70 kDa fitc-dextran tracer following treatment with 20% sera for 24 h; *n* = 10–11, * *p* < 0.05 *vs*. healthy donors.

Metabolite	Healthy donor (µM)	Hemodialysis (µM)	Permeability correlation	
			Rho	*P*_FDR_
*p*-Cresol sulfate	14.12 ± 3.09	155.26 ± 27.36 *	0.675	0.01
*p*-Cresol glucuronide	0.09 ± 0.03	10.63 ± 4.44 *	0.394	0.12
Indoxyl sulfate	2.92 ± 0.45	147.18 ± 26.46 *	0.400	0.09
Serotonin	0.64 ± 0.12	0.42 ± 0.18	−0.233	0.64
Kynurenine	1.73 ± 0.23	4.63 ± 0.57 *	0.371	0.06
3-Hydroxyanthranilic acid	0.01 ± 0.00	0.05 ± 0.01 *	0.434	0.11
Tryptophan	42.30 ± 1.46	22.97 ± 2.35 *	−0.466	0.06
5-Hydroxyindole acetic acid	0.05 ± 0.01	1.28 ± 0.21 *	0.464	0.08
Xanthurenic acid	0.01 ± 0.00	0.17 ± 0.05 *	0.442	0.02
Anthranilic acid	0.20 ± 0.02	0.52 ± 0.1 *	0.396	0.12
Kynurenic acid	0.06 ± 0.01	2.34 ± 0.58 *	0.353	0.16
Indole-3-lactic acid	0.78 ± 0.12	2.42 ± 0.33 *	0.397	0.12
Indole-3-carboxaldehyde	0.05 ± 0.00	0.06 ± 0.01	−0.08	0.78
Indole-carboxylic acid	0.04 ± 0.01	0.06 ± 0.01	0.041	0.89
Indole-3-acetic acid	2.45 ± 0.75	6.36 ± 2.07	0.385	0.17
Indole-3-propionic acid	1.73 ± 0.4	1.37 ± 0.35	0.051	0.47
Methyl indole 3-acetate	0.05 ± 0.03	7.44 ± 3.48 *	0.241	0.43

## Discussion

Impaired kidney function impacts virtually every organ system in the body, and the central nervous system is no exception. Patients with CKD, including those not yet requiring dialysis, exhibit significant cognitive decline^46^ and enhanced stroke risk,^47^ even after correction for typical cardiovascular risk factors. Here, we provide data indicating that the major uremic toxin and gut microbe–host, co-metabolite pCS, acts via the EGFR to mobilize MMP activity and damage the critically protective BBB, an effect known to underlie cerebrovascular risk.^48^ Moreover, we show that pCS treatment induces widespread transcriptomic changes within the brain, rapidly dampening numerous aspects of neuronal activity and metabolism. Together, these data strongly implicate pCS as a major contributory factor to the links between kidney failure and impaired brain function.

Interactions of pCS with the peripheral vasculature are known, with evidence of arterial leakage in patients with CKD^49^ and in animal^19^ and *in vitro* models of uremia,^50^ with uremic levels of pCS causing inter-endothelial adherens junction disruption.^49^ Here, we extend the damaging actions of pCS to the cerebral vasculature and the BBB, acting at concentrations similar to those of free pCS seen at stages III and IV of CKD.^51^ We further provide mechanistic evidence coupling pCS agonism at the EGFR, *via* phosphorylation of ANXA1 and STAT3, to mobilization of MMP activity, a known contributor to neurovascular disruption.^52^ Importantly, these actions appear clinically relevant, with the serum of human hemodialysis patients impairing *in vitro* brain endothelial permeability barrier both with a potency highly correlated to pCS content and with sensitivity to the EGFR antagonist erlotinib. Placing the EGFR as a major mediator of pCS actions in the brain additionally suggests potential therapeutic strategies to counter CKD-associated cerebrovascular damage; several EGFR inhibitors (e.g., erlotinib and gefitinib) and monoclonal antibodies (e.g., cetuximab and necitumumab) are licensed for clinical use in malignant disease, suggesting there may be scope for broader use in CKD, although caution is needed regarding the safety of at least some of these agents in advanced renal impairment.^53^ Nonetheless, this highlights the possibility of developing an alternative risk-reduction strategy for cerebrovascular complications in CKD, something currently chiefly addressed through management of hypertension.

Cerebral small vessel disease, for which BBB failure is a fundamental driver,^54^ is thought to contribute to both the cognitive impairments and enhanced stroke risk seen in CKD patients.^55^ What is less clear, partly due to the obscuring effects of the relationship between CKD and cerebrovascular risk factors such as diabetes and hypertension, is why this is the case. Activation of MMPs within the neurovascular unit has been reported in several forms of cerebrovascular disease, including stroke,^56^ traumatic injury,^57^ cerebral small vessel disease^58^ and, notably, in murine models of CKD.^59^ By elaborating a mechanistic link between pCS, cerebrovascular MMP activity, and impaired BBB integrity, we provide an explanation for how cerebral vessels may be damaged in CKD. Moreover, whilst local MMP activity has been demonstrated in a range of kidney disorders,^60^ whether this holds true in the brains of CKD patients is unknown. Our data indicating a close correlation between pCS content and the BBB-permeabilizing actions of hemodialysis patient serum and that exposure to such serum directly enhances endothelial MMP expression suggest that this is highly likely.

While enhanced BBB permeability is a recognized driver of damage to cognitive function,^61^ our data suggest that the effects of pCS upon brain function extend beyond its immediate impact on the cerebral vasculature. Transcriptional analysis of pCS-exposed mice indicates a more generalized inhibitory effect of the toxin upon neural activity, which might also be expected to impair cognitive abilities, although it is not possible to generalize these changes to the effects of long-term pCS exposure. Despite these links, we did not detect such cognitive disruption following long-term pCS exposure, however, a finding with several potential explanations. Previous studies identifying cognitive sequelae of BBB damage have shown a substantial increase in permeability, in the order of a 5-fold increase or greater,^62,63^ to be associated with cognitive deficits. In our study, animals exposed for one month to pCS exhibited an approximately 30% increase in tracer permeability across the BBB; it may be that the degree of permeability deficit caused in this study was insufficient to impair cognition to a degree detectable using the tests employed. Notably, previous studies employing higher pCS concentrations have shown the metabolite to impair cognition in both humans^64^ and mice.^62^

Intriguingly, while pCS clearly influenced BBB permeability, in our hands, this was not the case for the other major gut microbe-derived uremic toxin, IS. This is perhaps surprising, given previous evidence that IS concentrations correlate with BBB impairment in partially nephrectomized rats^62^ and vascular disease in CKD patients.^65^ In contrast, however, suppression of raised circulating IS by sequestration did not reverse adenine-rich diet-induced BBB impairment,^62^ highlighting the complexity of the pathways impinging on the BBB. Taken alongside our recent demonstration that the CKD-associated molecule trimethylamine *N*-oxide (TMAO) exerted marked beneficial effects upon both the BBB and cognition at levels seen in metabolically healthy individuals,^22^ it appears the broad assumption that uremia-associated molecules are always deleterious may need revisiting.^66^

While pCS predominates in the circulation, it is not the only *p*-cresol conjugate found in blood, with pCG representing a significant, albeit minor, fraction.^12^ Moreover, levels of pCG do increase markedly with worsening CKD stages, and have been shown to correlate with mortality risk in a similar way to pCS.^67^ However, we have shown pCG to exert very different actions upon the BBB to pCS, with its administration at physiologically relevant levels *in vivo* enhancing barrier integrity by antagonism of basal circulating lipopolysaccharide interactions with TLR4.^68^ Moreover, whole-brain RNAseq analysis of pCG-exposed mice indicated a positive regulation in neuronal activity gene ontologies, notably opposite to the suppressive effects of pCS described here.^68^ That the two major conjugates of gut microbe-derived *p*-cresol should so markedly differ in effect upon the brain is striking, emphasizing the need for mechanistic studies of gut microbe/host co-metabolite action – and in differing contexts (i.e. healthy vs disease) – before conclusions can safely be drawn about their clinical role(s).

## Conclusions

Most circulating pCS is ultimately derived from the actions of gut microbes upon dietary amino acids,^8^ thus our description of its effects upon the BBB further emphasizes the importance of this facet of gut microbe–host interactions. Notably, while we and others have shown other gut microbe-derived metabolites, including short-chain fatty acids,^30,69^ methylamines,^22^ and pCG,^68^ to reinforce BBB integrity in healthy animals, pCS is overtly detrimental in its effects. Given the wide range of metabolites produced by gut microbes, the fact that some agents are deleterious at supraphysiological concentrations is unsurprising, but this does emphasize the importance of mechanistic analyses of gut-brain communication pathways. Overall, our description of pCS as a major link between gut microbes, kidney function, and the cerebral vasculature highlights the role of the gut microbiota in at least some of the systemic effects of renal disease, emphasizing its potential as a therapeutic target for clinical management of CKD.

## List of abbreviations

**Table:** 

BBB	blood – brain barrier
CKD	chronic kidney disease
EGFR	epidermal growth factor receptor
IS	indoxyl sulfate
MMP	matrix metalloproteinase
Pcs	*p*-cresol sulfate
pCG	*p*-cresol glucuronide
SPIA	signalling pathway impact analysis
TEER	trans-endothelial electrical resistance
ZO-1	zonula occludens-1

## Ethics approval and consent to participate

Use of human serum samples was approved by the Barts Diabetic Kidney Disease Biobank under Barts Health NHS Trust Research Ethics Committee reference 20/LO/0361. All animal work was approved by either the Queen Mary University of London or University of East Anglia Animal Welfare Ethical Review Boards as indicated in the relevant experimental methods sections.

## Availability of data and material

Raw RNAseq data have been deposited with ArrayExpress under the accession number E-MTAB-12326. This paper does not report the original code. All other data are included in the manuscript or supplementary files.

## Supplementary Material

Supplemental Material
