# Supplementary material for: Cerebrovascular damage caused by the gut microbe/host co-metabolite p-cresol sulfate is prevented by blockade of the EGF receptor
Source: Gut Microbes. 2024 Nov 24;16(1):2431651. doi: 10.1080/19490976.2024.2431651 (PMC11591591; doi:10.1080/19490976.2024.2431651)
Supplement: Supplemental Material [file KGMI_A_2431651_SM8359.zip › Supplemental_Figures_clean.docx]

1. *P*<0.001


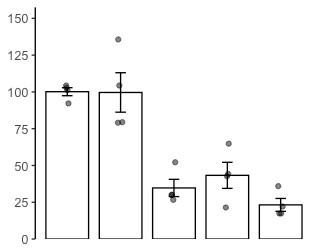


Cell surface EGF

-

R expression

(

)

% of mock transfected cells

*P*

<0.001

*P*

<0.001


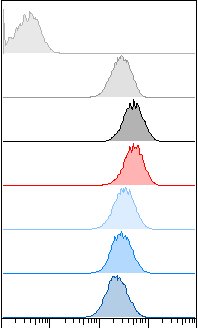


Unstained

2

nd

Antibody only

Mock transfected

Negative control siRNA

siRNA 5

siRNA 11

siRNA 12

| Mock -ve siRNA siRNA siRNA siRNA 5 11 12 | 10^0^ 10^1^ 10^2^ 10^3^ 10^4^  Fluorescence intensity |
| --- | --- |


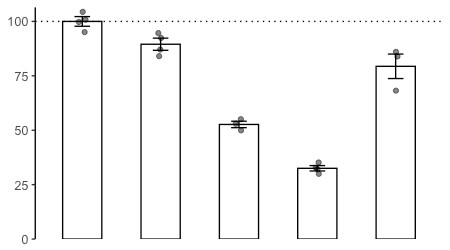


Total ANXA1 expression

% of wild

(

-

type)

P<0.001

P<0.001

P=0.001

Unstained

2

nd

Ab

Wild

-

type

Scramble

Clone 60A

Clone 60B

Clone 57/61


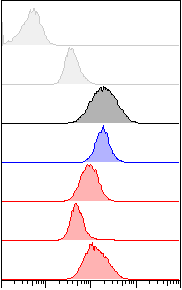


Wild-type Scramble 60A 60B 57/61 10^0^ 10^1^ 10^2^ 10^3^ 10^4^

**Supplemental Figure 1: Confirmation of RNA interference knock-down experiments.** A) Transfection of hCMEC/D3 cells with siRNA sequences targeting EGFR causes a significant downregulation of expression 96 h later when compared with mock transfected cells or cells transfected with a non-targeting negative control siRNA sequence; data are mean ± s.e.m., n = 4, representative flow cytometry histograms are shown. B) Stable transfection of hCMEC/D3 cells with shRNA sequences targeting ANXA1 causes a reduction in protein expression, an effect not seen upon transfection with a scramble shRNA sequence; data are expressed as mean ± s.e.m., *n*=4 independent experiments, representative flow cytometry histograms are shown.

B

C


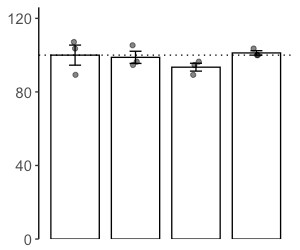


Transendothelial electrical

resistance (% of control)

Control

10

µM

µM

100

mM

1

Control

µM

10


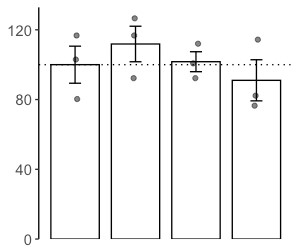


µM

100

1

mM

Paracellular permeability to a 70kDa

FITC

-

dextran tracer (% of control)

0

µM

µM

10

µM

100

mM

1

pCS


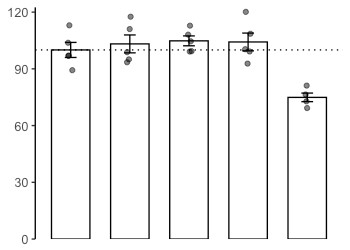


%

0.03

H

2

O

2

Cell survival (% of untreated)

*P*

=0.002

A

**Supplemental Figure 2: Confirmation of specificity of pCS effects upon endothelial barrier function in hCMEC/D3 cells.** A) Treatment of hCMEC/D3 cells with increasing concentrations of pCS (10 µM, 100 µM, 1 mM, 24 h) does not affect cell viability as assessed by MTT assay, in contrast to treatment with 0.03 % H_2_O_2_ (24 h), which caused a significant loss in viability; data are mean ± s.e.m., n = 5 independent experiments. B) Incubation of hCMEC/D3 cell monolayers with KCl (10 µM - 1 mM; 24 h) did not significantly affect paracellular permeability to a 70 kDa FITC-dextran conjugate; data are mean ± s.e.m., n = 3 independent experiments. C) Incubation of hCMEC/D3 cell monolayers with KCl (10 µM - 1 mM; 24 h) did not significantly affect TEER; data are mean ± s.e.m., n = 3 independent experiments.


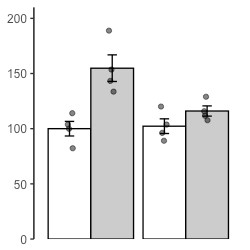


Untreated

Albumin

Control

pCS

*P*

=0.002

*P*

=0.022

Paracellular permeability to a 70kDa

FITC

-

dextran tracer (% of control)


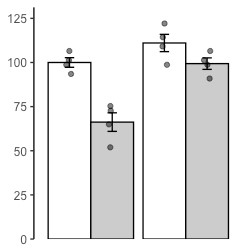


Untreated

Albumin

Control

pCS

*P*

<0.001

*P*

<0.001

Transendothelial electrical

resistance (% of control)

A

B

**Supplemental Figure 3: Treatment of hCMEC/D3 cells with albumin prevented the effects of pCS upon endothelial barrier function.** A) Incubation of hCMEC/D3 cell monolayers with human albumin (50 mg/ml) in conjunction with pCS (10 µM, 24 h) prevented the effects of pCS upon paracellular permeability to a 70 kDa FITC-dextran conjugate; data are mean ± s.e.m., n = 4 independent experiments. B) Incubation of hCMEC/D3 cell monolayers with human albumin (50 mg/ml) in conjunction with pCS (10 µM, 24 h) prevented the effects of pCS upon trans-endothelial electrical resistance; data are mean ± s.e.m., n = 4 independent experiments.

1. Saline pCS


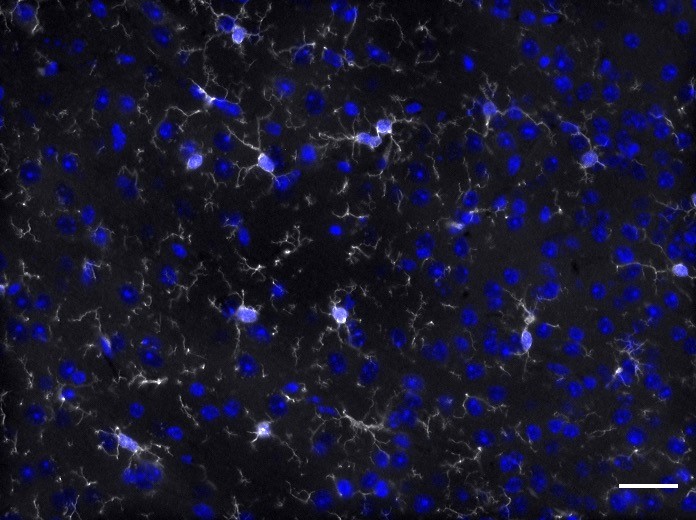

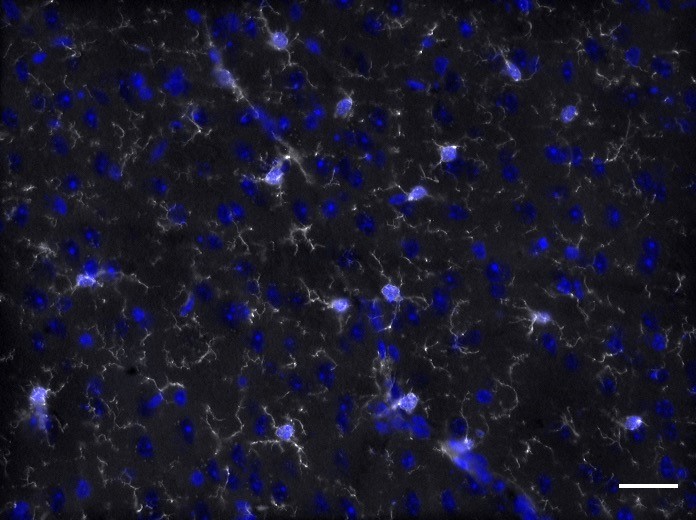


Iba1

DAPI

Iba1

DAPI

1. Saline pCS


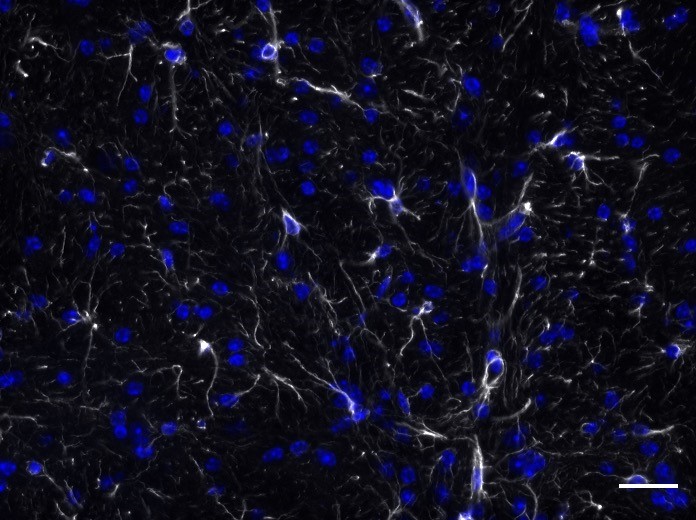

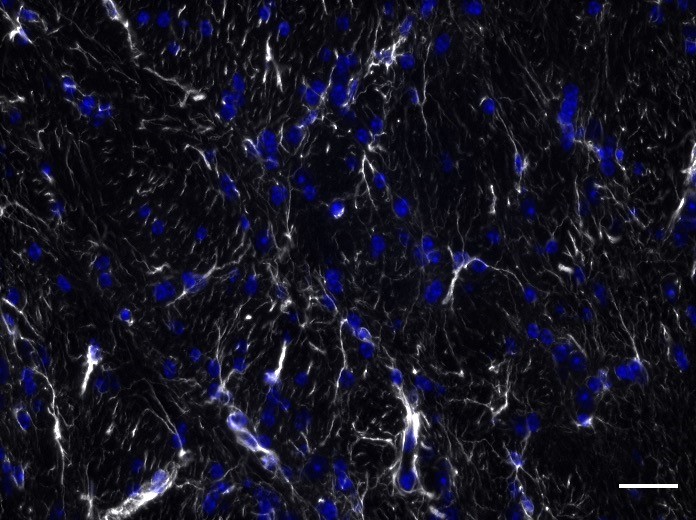


GFAP

DAPI

GFAP

DAPI

**Supplemental Figure 4: Mice exposed to raised levels of pCS for 4 weeks do not show any obvious changes in cortical microglial or astrocyte morphology or density.** Typical immunofluorescent analysis of A) Iba1 expression (microglia) or B) GFAP expression (astrocytes) within the cerebral cortex of male C57Bl/6 mice implanted with minipumps designed to release saline vehicle (n=10) or pCS (n=7) continuously for four weeks. Nuclei are counterstained with DAPI (blue), scale bar = 30 µm.

A B C


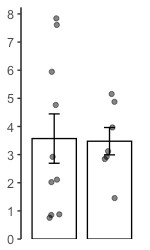


Time in centre of field (%)


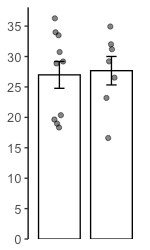


Distance travelled (m)


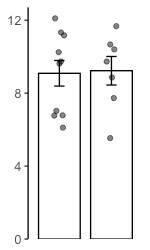


Mean velocity (cm/s)

Saline pCS Saline pCS Saline pCS

D E F


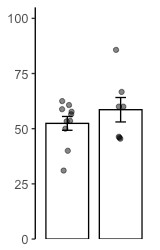


Spontaneous alternation (%)

Distance travelled (m)


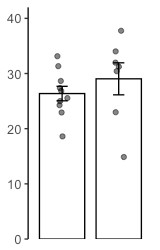

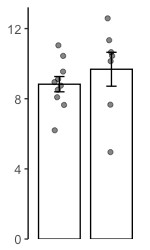


Mean velocity (cm/s)

Saline pCS Saline pCS Saline pCS

G


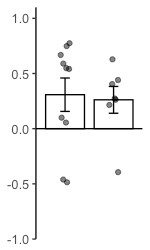


Discrimination index

Saline pCS

**Supplemental Figure 5: Mice exposed to raised levels of pCS for 4 weeks do not show overt behavioural changes on the open field test, Y-maze or novel object recognition task.** Mice implanted with minipumps designed to release saline vehicle or pCS continuously for four weeks were assessed for A) percentage of time in the centre zone, B) distance travelled and C) movement speed in the open field test, D) spontaneous alteration, E) distance travelled and F) mean velocity in the Y-maze task and G) discrimination index in the novel object recognition test. Data are expressed as mean ± s.e.m., *n* = 7-10 mice.


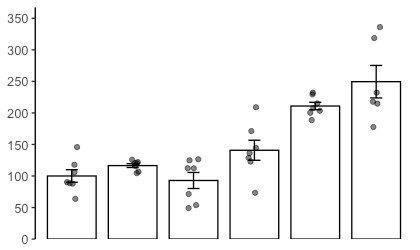


0

µM

1

µM

10

µM

100

µM

1

mM

1

µM

Rotenone

ROS production rate

(

)

% of untreated

*P*

<0.001

*P*

<0.001

pCS

**Supplemental Figure 6: pCS exposure induced oxidative stress in hCMEC/D3 cells only at high concentrations.** Treatment of hCMEC/D3 cells with pCS stimulated reactive oxygen species production at 1 mM, but not at lower concentrations (1 µM, 10 µM, 100 µM); the mitochondrial complex I inhibitor rotenone (1 µM) was used as a positive control, data are mean ± s.e.m., n=7 independent experiments.


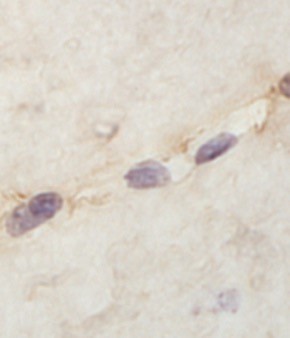

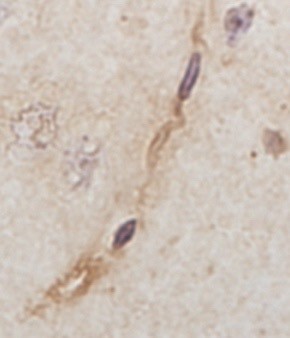

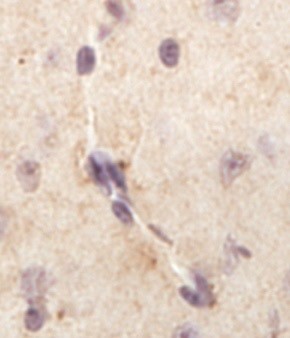

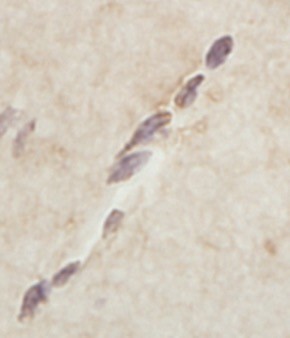


Saline

pCS

Saline

Erlotinib


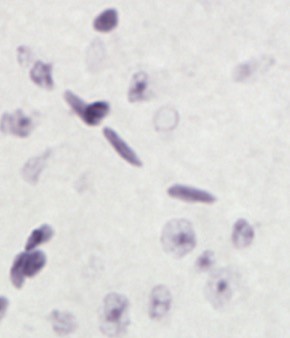


2

nd

Ab only

**Supplemental Figure 7: *In vivo* administration of pCS stimulates Tyr21 phosphorylation of ANXA1 in an erlotinib-sensitive manner.** Typical immunohistochemical analysis of Tyr21phosphorylated ANXA1 expression within the cerebral microvasculature of male C57Bl/6 mice treated with pCS (10 mg/kg, i.p. 2 h) with or without erlotinib pre-treatment (50 mg/kg, i.p. 1 h pre-treatment); nuclei are counterstained with haematoxylin, scale bar = 15 µm.


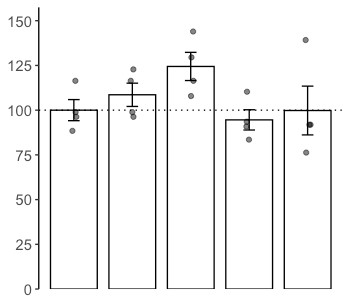


Paracellular permeability to a 70kDa

FITC

-

dextran tracer (% of control)

Control

A

5

µM

25

µM

50

µM

150

µM


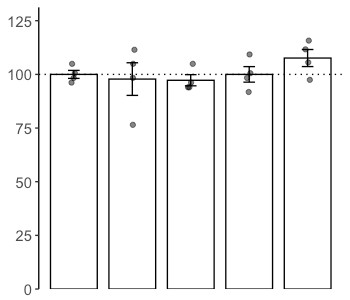


Transendothelial electrical

resistance (% of control)

B

Control

5

µM

25

µM

50

µM

150

µM

IS IS

**Supplemental Figure 8: Treatment of hCMEC/D3 cells with indoxyl sulfate (IS) does not affect endothelial barrier functions at physiological or uremia-associated concentrations.** A) Incubation of hCMEC/D3 cell monolayers with IS (5 µM, 25 µM, 50 µM, 150 µM; 24 h) did not significantly affect paracellular permeability to a 70 kDa FITC-dextran conjugate at any concentration tested; data are mean ± s.e.m., n = 4 independent experiments. B) Incubation of hCMEC/D3 cell monolayers with IS (5 µM, 25 µM, 50 µM, 150 µM; 24 h) did not significantly affect TEER at any concentration tested; data are mean ± s.e.m., n = 4 independent experiments.


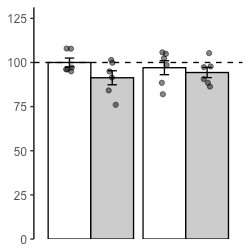


Transendothelial electrical

resistance (% of control)

Control

Erlotinib

Paracellular permeability to a 70kDa

FITC

-

dextran tracer (% of control)

Control

Erlotinib

A

B


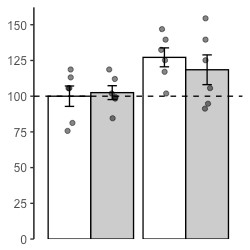


*P*

<0.01

Untreated XA Untreated XA

(0.2 µM) (0.2 µM)

**Supplemental Figure 9: Treatment of hCMEC/D3 cells with xanthurenic acid (XA) increased endothelial barrier function at uremia-associated concentrations, in an erlotinib-insensitive manner.** A) Incubation of hCMEC/D3 cell monolayers with XA (0.2 µM; 24 h) significantly increased paracellular permeability to a 70 kDa FITC-dextran conjugate, but this was insensitive to erlotinib pretreatment (2.5 µM, 10 minutes prior to XA); data are mean ± s.e.m., n = 6 independent experiments. B) Incubation of hCMEC/D3 cell monolayers with XA (0.2 µM; 24 h) did not significantly affect TEER, nor was there any significant effect of erlotinib pre-treatment (2.5 µM, 10 minutes prior to XA); data are mean ± s.e.m., n = 6 independent experiments.
