# Supplementary material for: Cerebrovascular damage caused by the gut microbe/host co-metabolite p-cresol sulfate is prevented by blockade of the EGF receptor
Source: Gut Microbes. 2024 Nov 24;16(1):2431651. doi: 10.1080/19490976.2024.2431651 (PMC11591591; doi:10.1080/19490976.2024.2431651)
Supplement: Supplemental Material [file KGMI_A_2431651_SM8359.zip › Supplemental_table_2.docx]

**Supplemental Table 2**: Circulating concentrations (µM) of *p*-cresol and tryptophan metabolites in mice exposed for 4 weeks to pCS or saline *via* osmotic minipumps. Data are mean ± s.e.m., n=7-10, * indicates statistical significance at *P*<0.05 or less

|  | **Control** | **pCS treated** | ***P*** |
| --- | --- | --- | --- |
| p-Cresol sulfate | 11.37 ± 0.89 | 38.75 ± 12.91 | 0.021* |
| p-Cresol glucuronide | 1.91 ± 0.32 | 1.21 ± 0.59 | 0.277 |
| Kynurenine | 1.57 ± 0.09 | 1.43 ± 0.09 | 0.291 |
| Tryptophan | 44.62 ± 2.37 | 40.76 ± 3.29 | 0.344 |
| Indoxyl sulfate | 7.51 ± 1.20 | 7.67 ± 1.74 | 0.938 |
| Anthranilic acid | 0.20 ± 0.05 | 0.16 ± 0.03 | 0.590 |
| Kynurenic acid | 0.21 ± 0.03 | 0.26 ± 0.09 | 0.582 |
| Indole-3-acetic acid | 0.31 ± 0.06 | 0.41 ± 0.09 | 0.352 |
| Indole-3-propionic acid | 0.64 ± 0.13 | 0.71 ± 0.15 | 0.726 |
| Indole-3-lactic acid | 1.77 ± 0.27 | 2.37 ± 0.55 | 0.300 |
